# Supplementary figures and images for: Canonical and phosphoribosyl ubiquitination coordinate to stabilize a proteinaceous structure surrounding the Legionella-containing vacuole
Source: eLife. 2026 Jul 8;14:RP108254. doi: 10.7554/eLife.108254 (PMC13345631; doi:10.7554/eLife.108254)

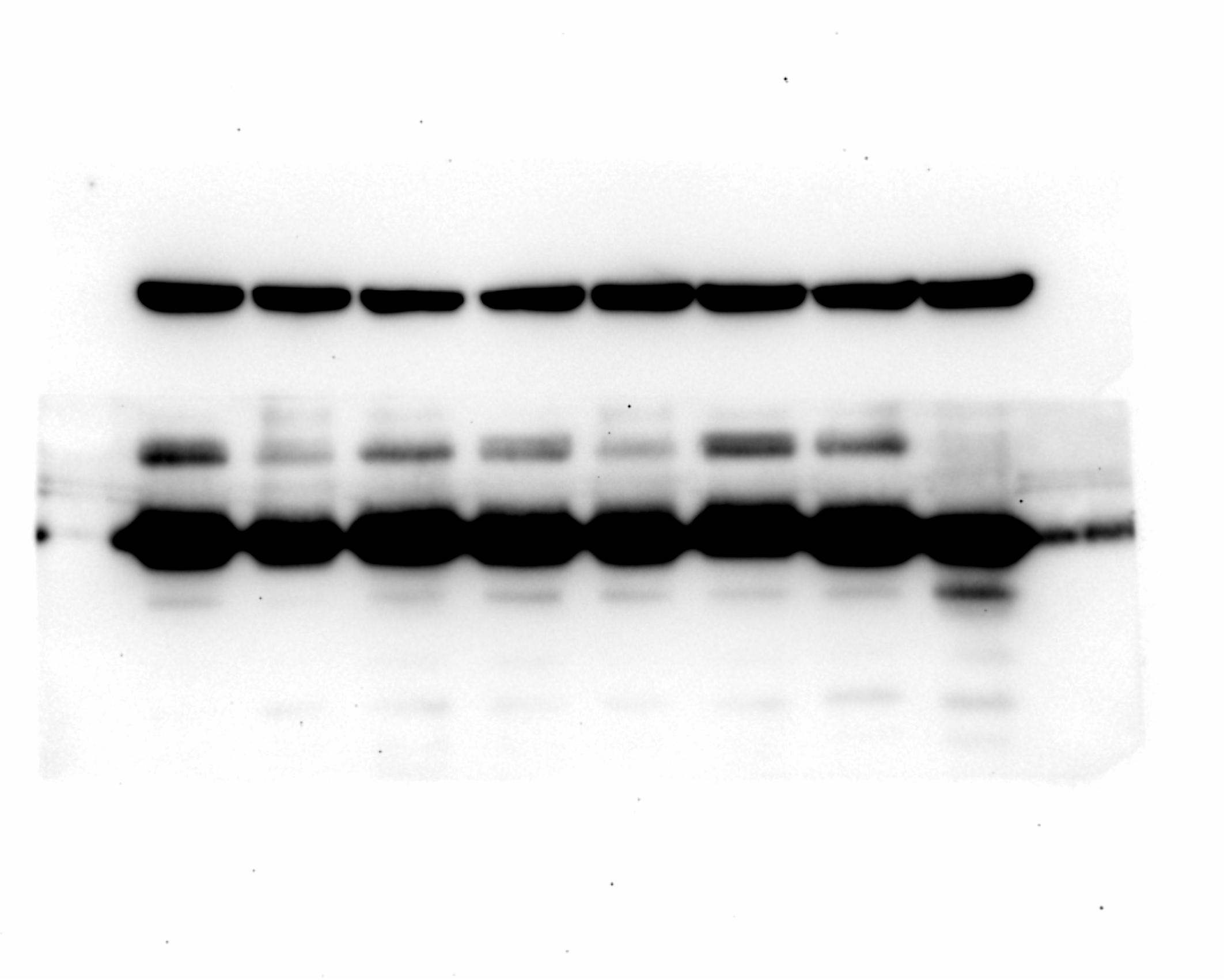

Supplement: Figure 1—figure supplement 1—source data 1. [file elife-108254-fig1-figsupp1-data1.zip › Figure 1, figure supplement 1 - source data 1/1s1C_1_gel1_flag_hsp70.tif]

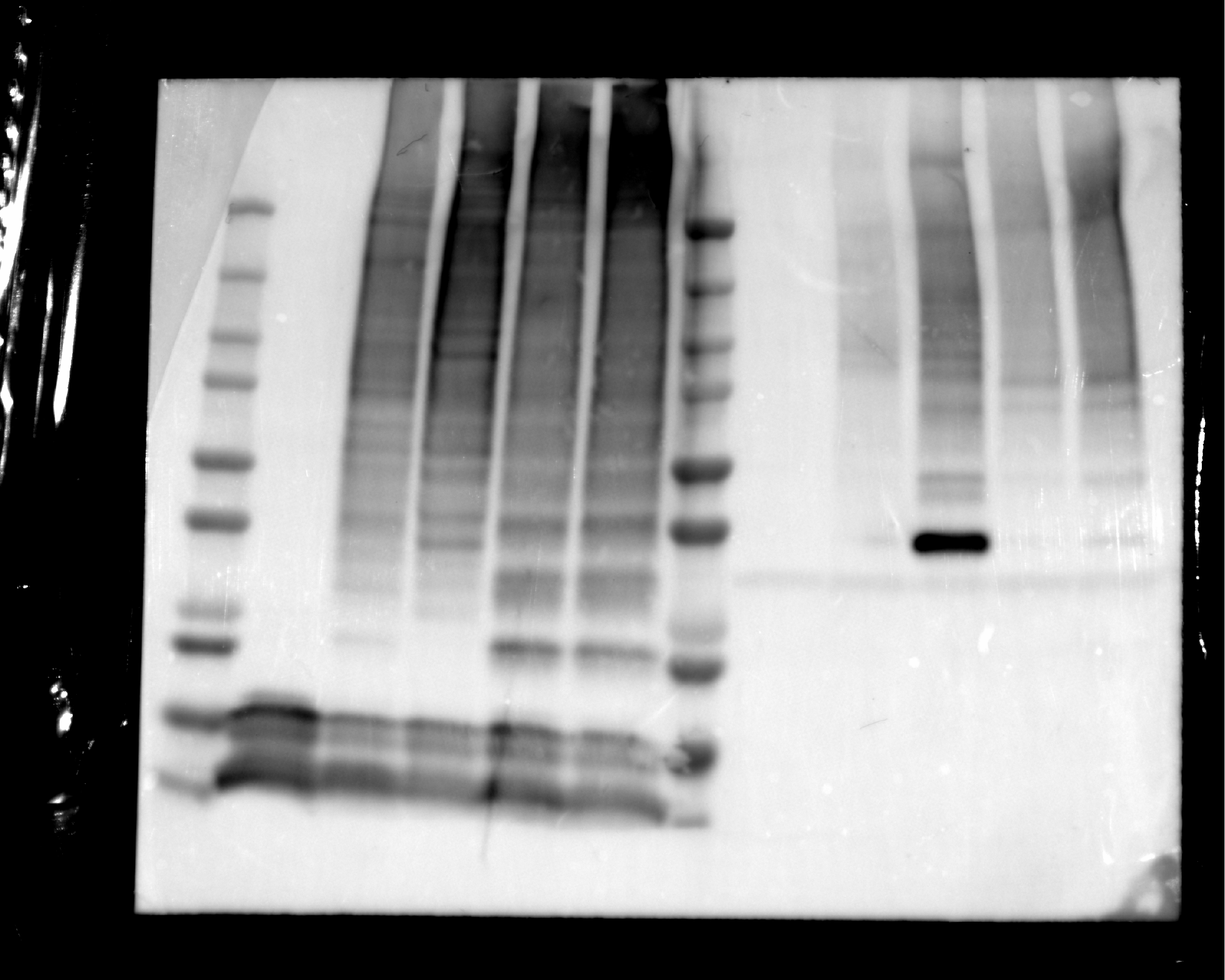

Supplement: Figure 2—source data 2. [file elife-108254-fig2-data2.zip › Figure 2 - source data 1/2c_1_20250317_MET093_HAHRP_300s_laddermerge.tif]

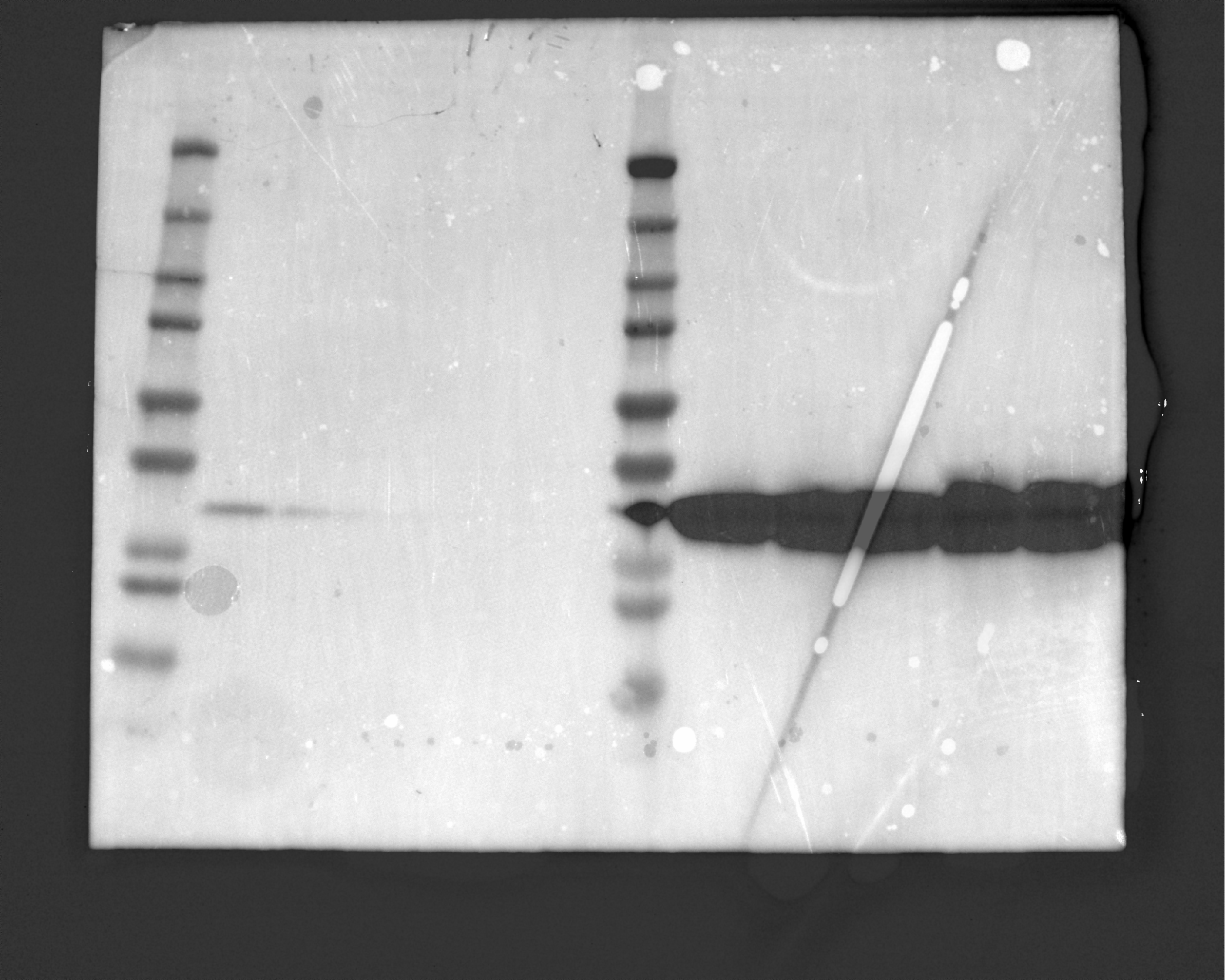

Supplement: Figure 2—source data 2. [file elife-108254-fig2-data2.zip › Figure 2 - source data 1/2c_2_20250324_MET093_Flag_ladder_merge.tif]

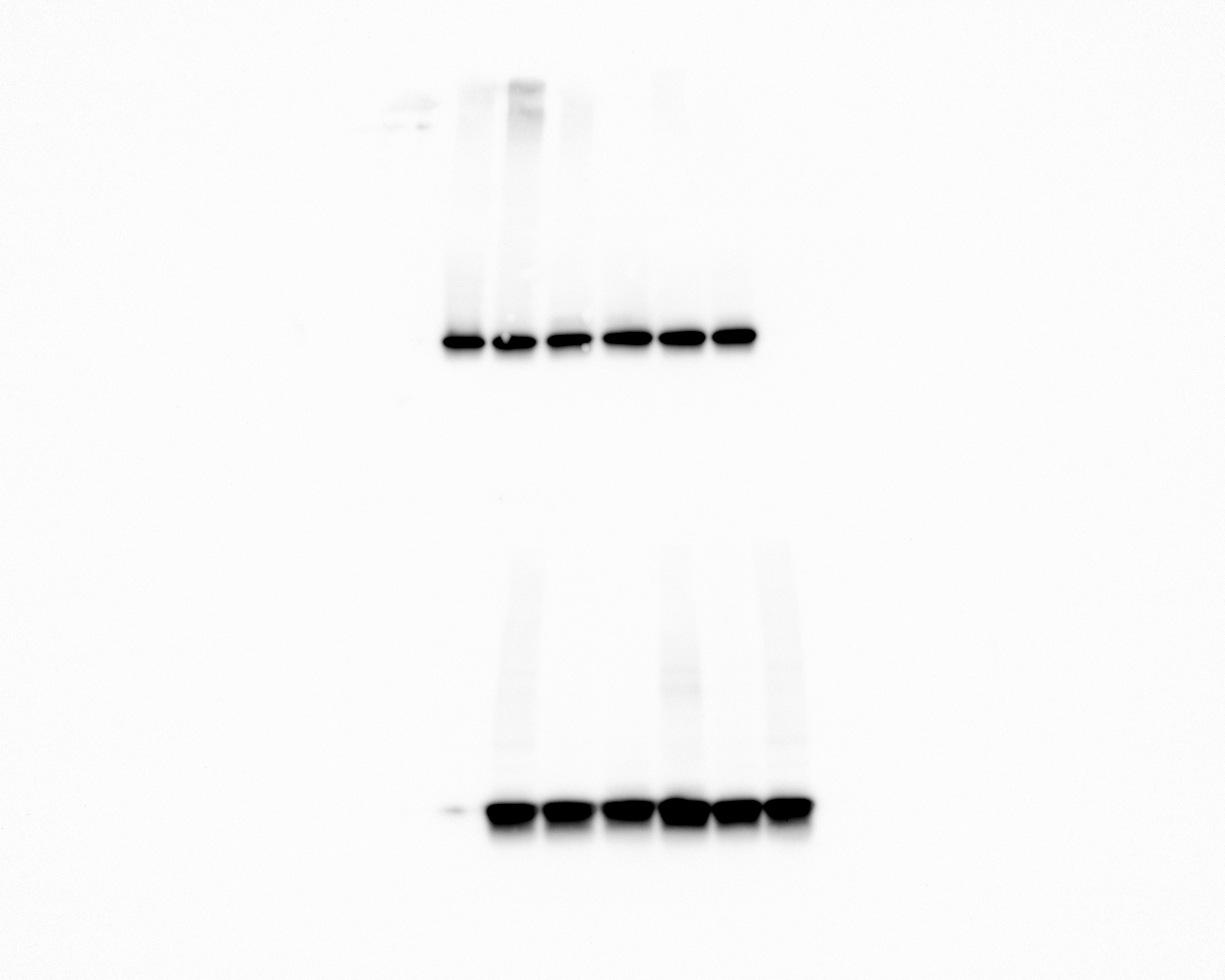

Supplement: Figure 2—source data 2. [file elife-108254-fig2-data2.zip › Figure 2 - source data 1/2d_3_20250331_MET099_Flag_InputIP_300s.tif]

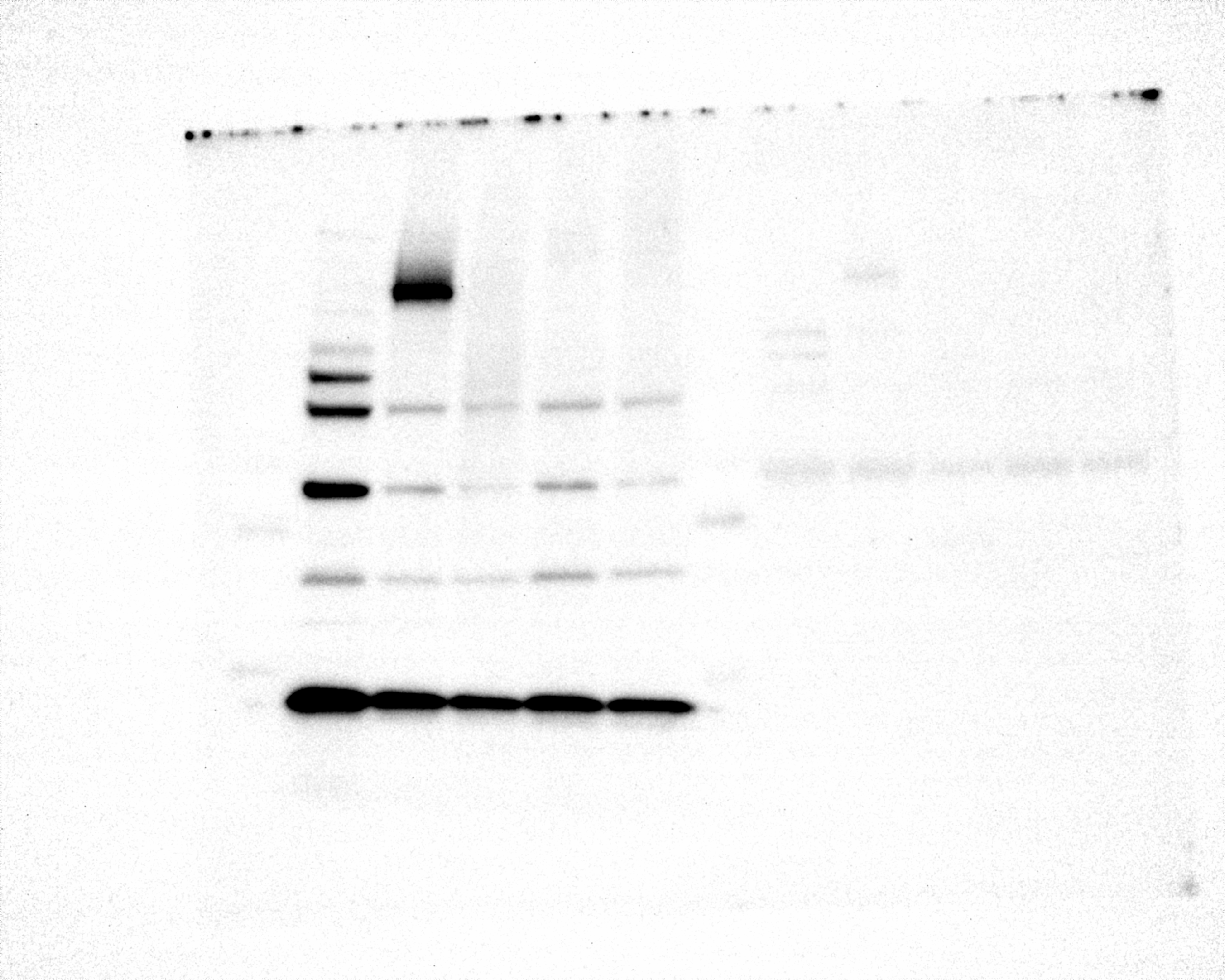

Supplement: Figure 2—source data 2. [file elife-108254-fig2-data2.zip › Figure 2 - source data 1/2c_3_20250318_MET093_Myc_300s_HC.tif]

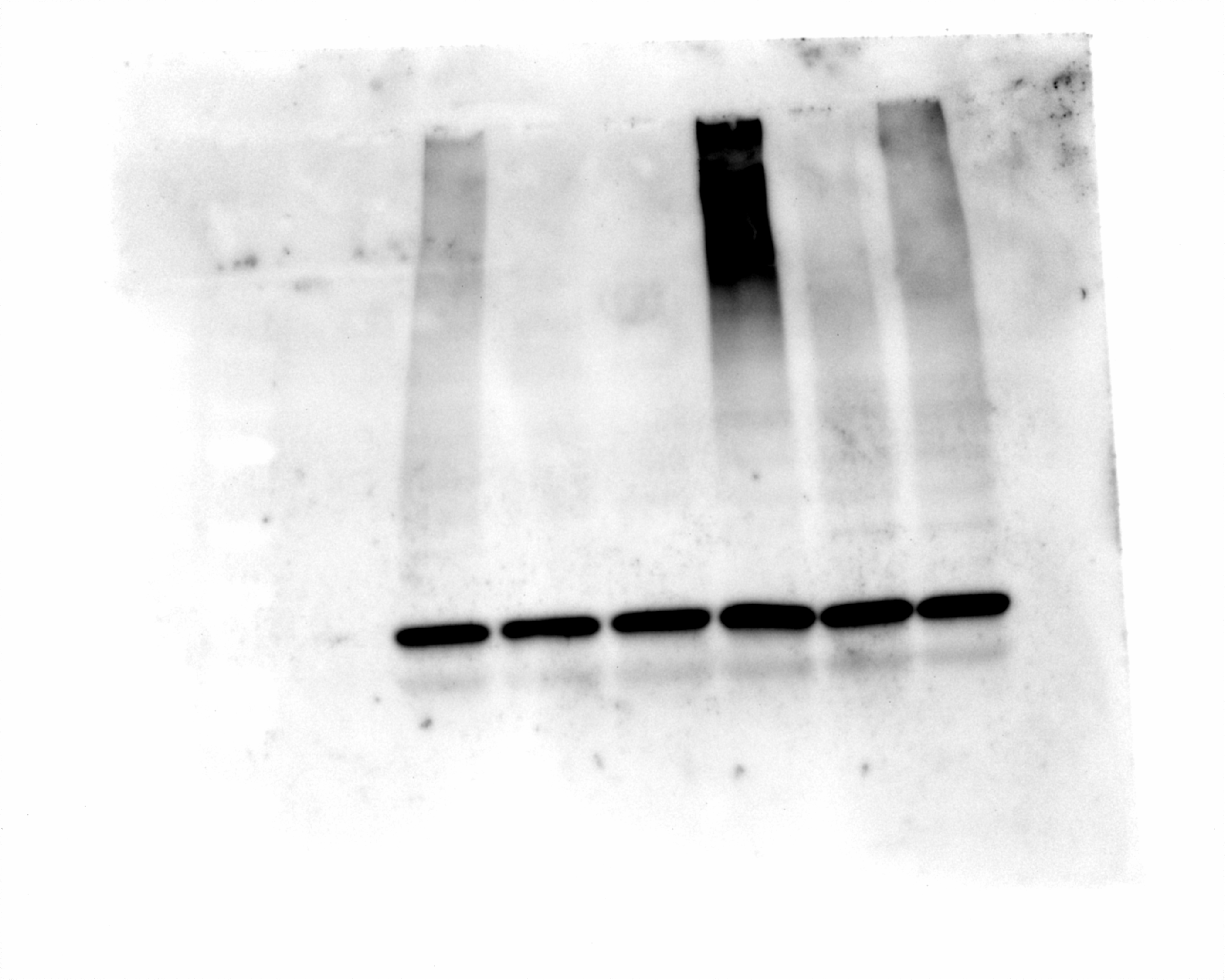

Supplement: Figure 2—source data 2. [file elife-108254-fig2-data2.zip › Figure 2 - source data 1/2d_2_20250330_MET099_IP_HAHRP_600s_Crescendo.tif]

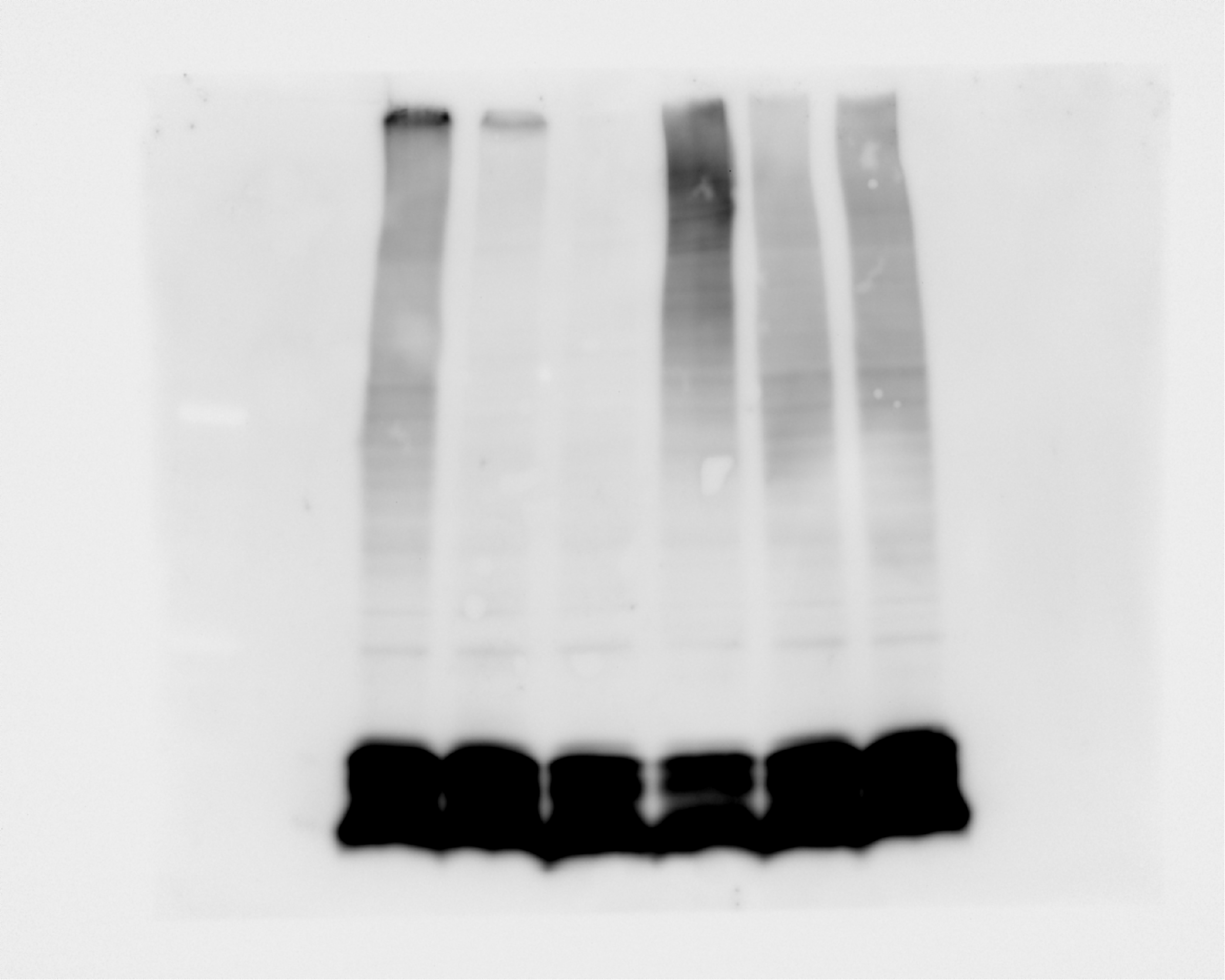

Supplement: Figure 2—source data 2. [file elife-108254-fig2-data2.zip › Figure 2 - source data 1/2d_1_20250330_MET099_Input_HAHRP_141s_Crescendo.tif]

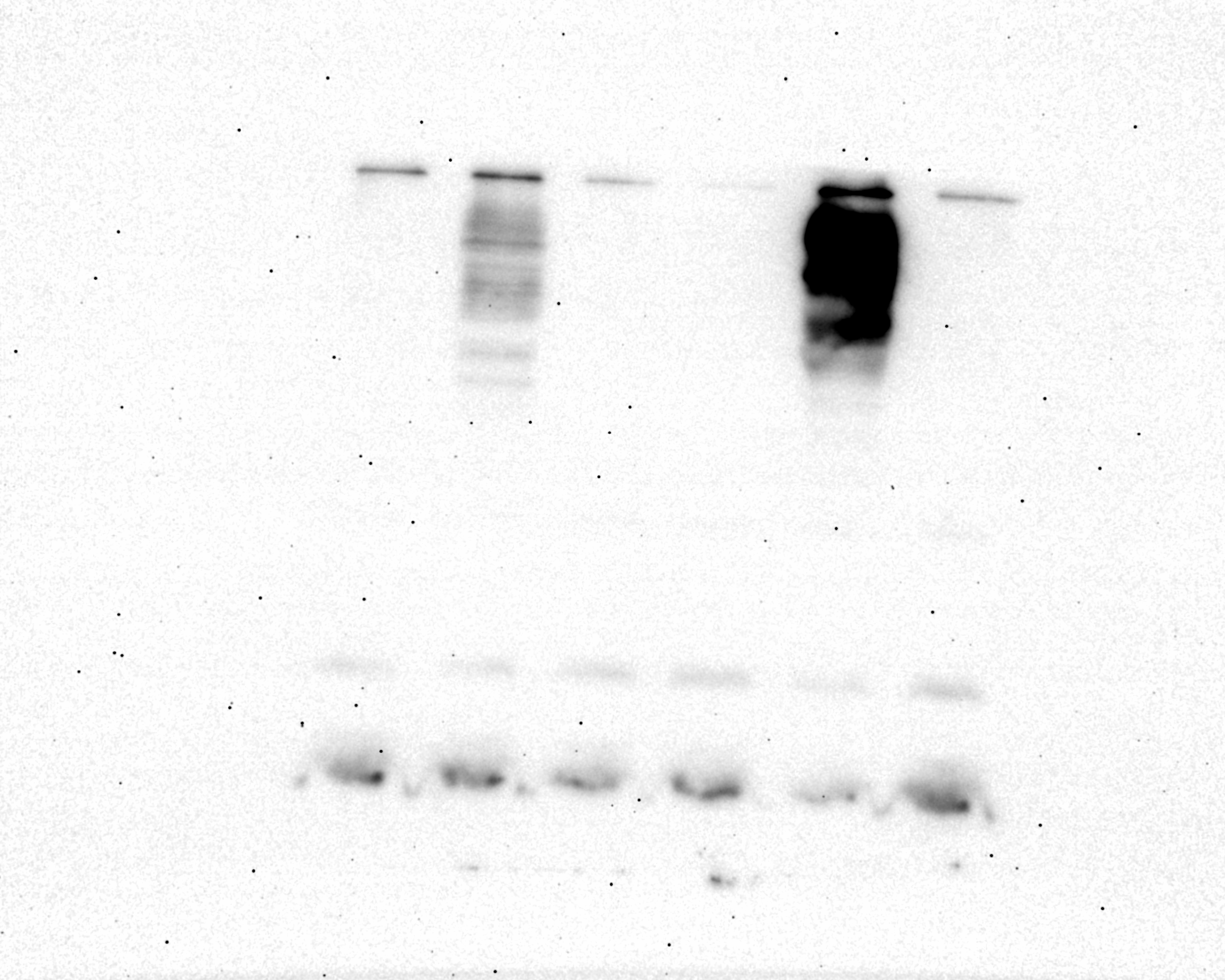

Supplement: Figure 2—figure supplement 1—source data 1. [file elife-108254-fig2-figsupp1-data1.zip › Figure 2, figure supplement 1 - source data 1/2s1_1_20240205_HAdGG_337s_HC.tif]

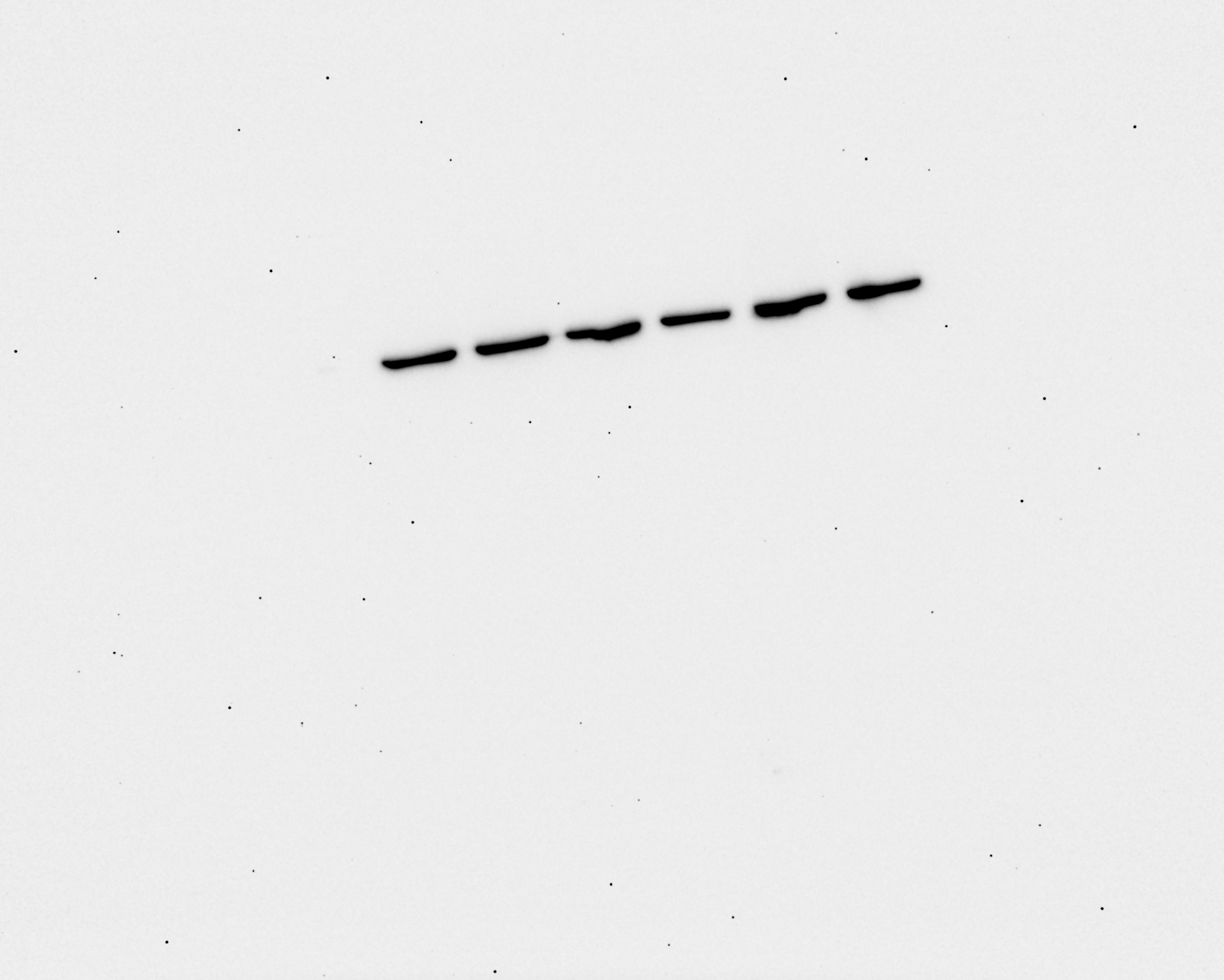

Supplement: Figure 2—figure supplement 1—source data 1. [file elife-108254-fig2-figsupp1-data1.zip › Figure 2, figure supplement 1 - source data 1/2s1_2_20240206_Hsp70_75s_HC.tif]

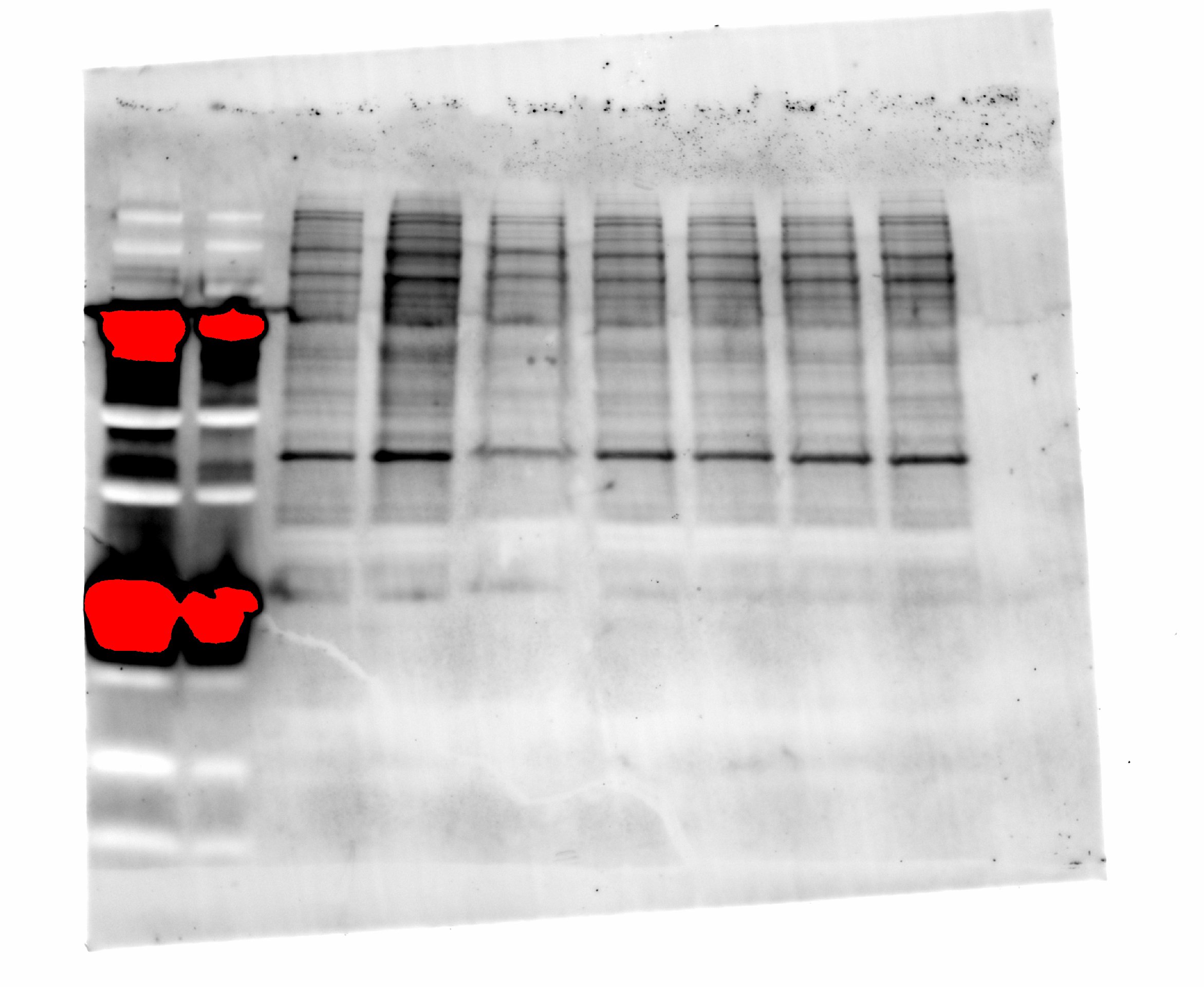

Supplement: Figure 5—source data 3. [file elife-108254-fig5-data3.zip › Figure 5 - source data 1/5e_1_20241007_MET071_StainFree_Insol.tif]

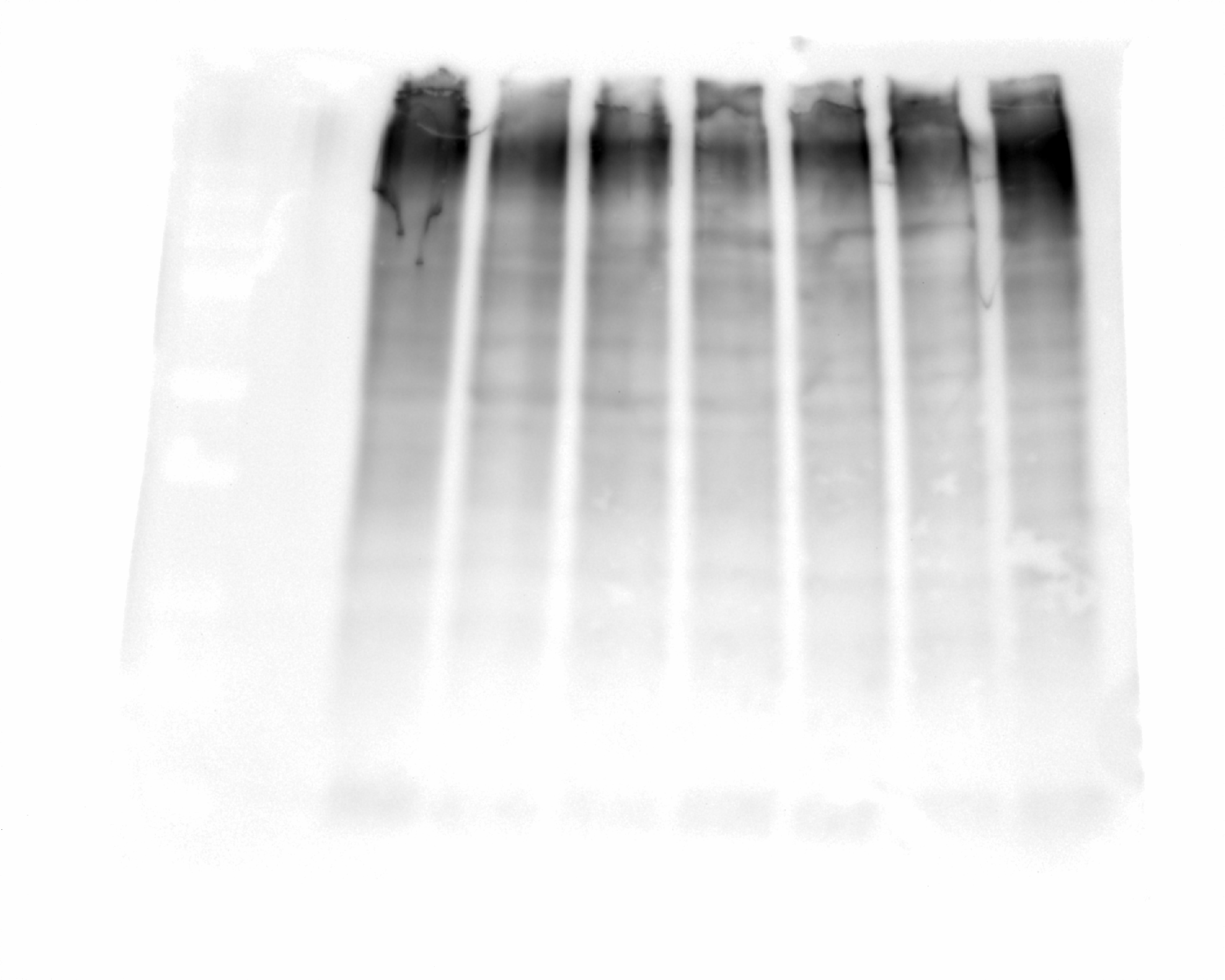

Supplement: Figure 5—source data 3. [file elife-108254-fig5-data3.zip › Figure 5 - source data 1/5g_1_SidCSdcA Rep3 SOLUBLE P4D1.tif]

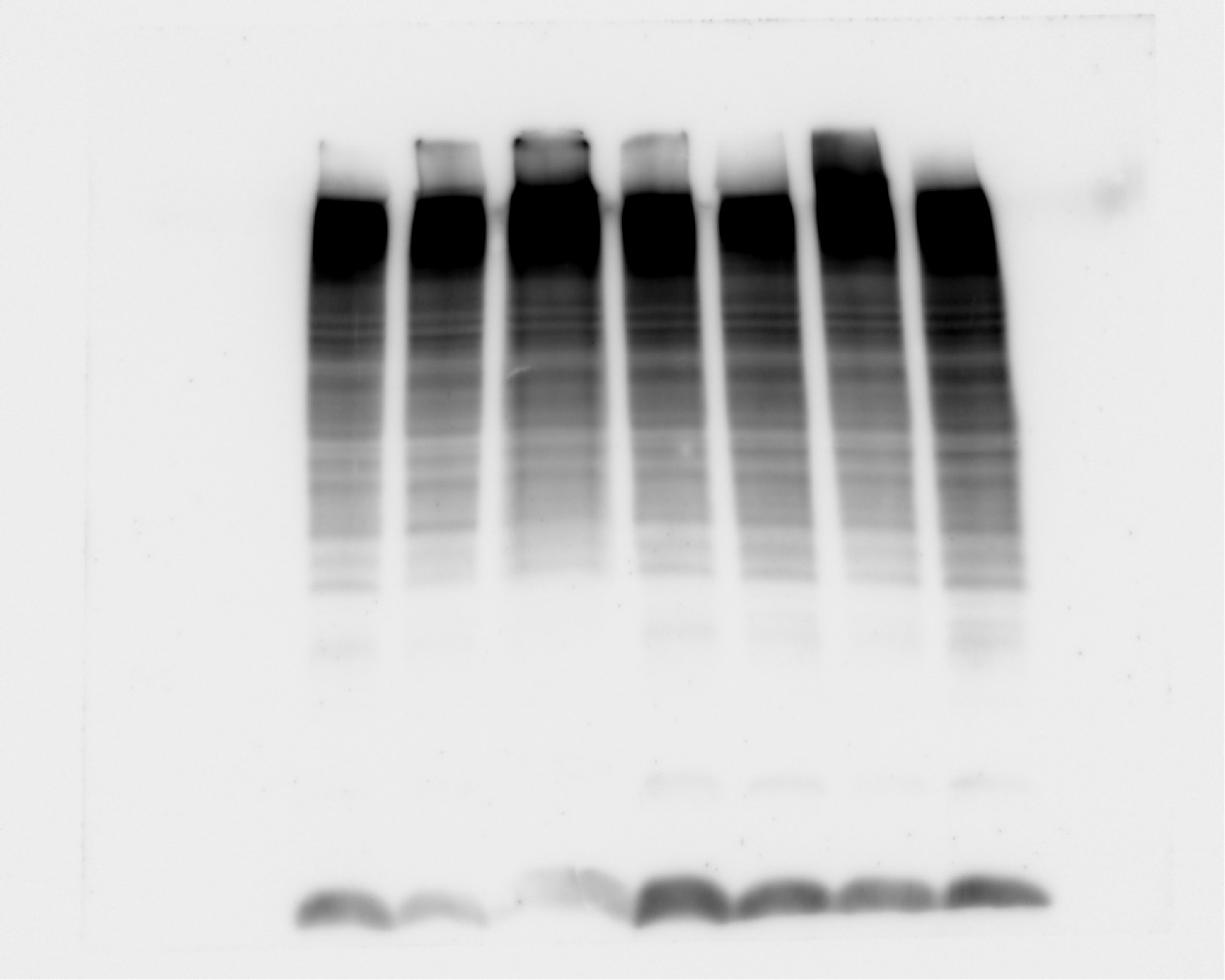

Supplement: Figure 5—source data 3. [file elife-108254-fig5-data3.zip › Figure 5 - source data 1/5d_1_20241007_MET071_Sol_P4D1_68s.tif]

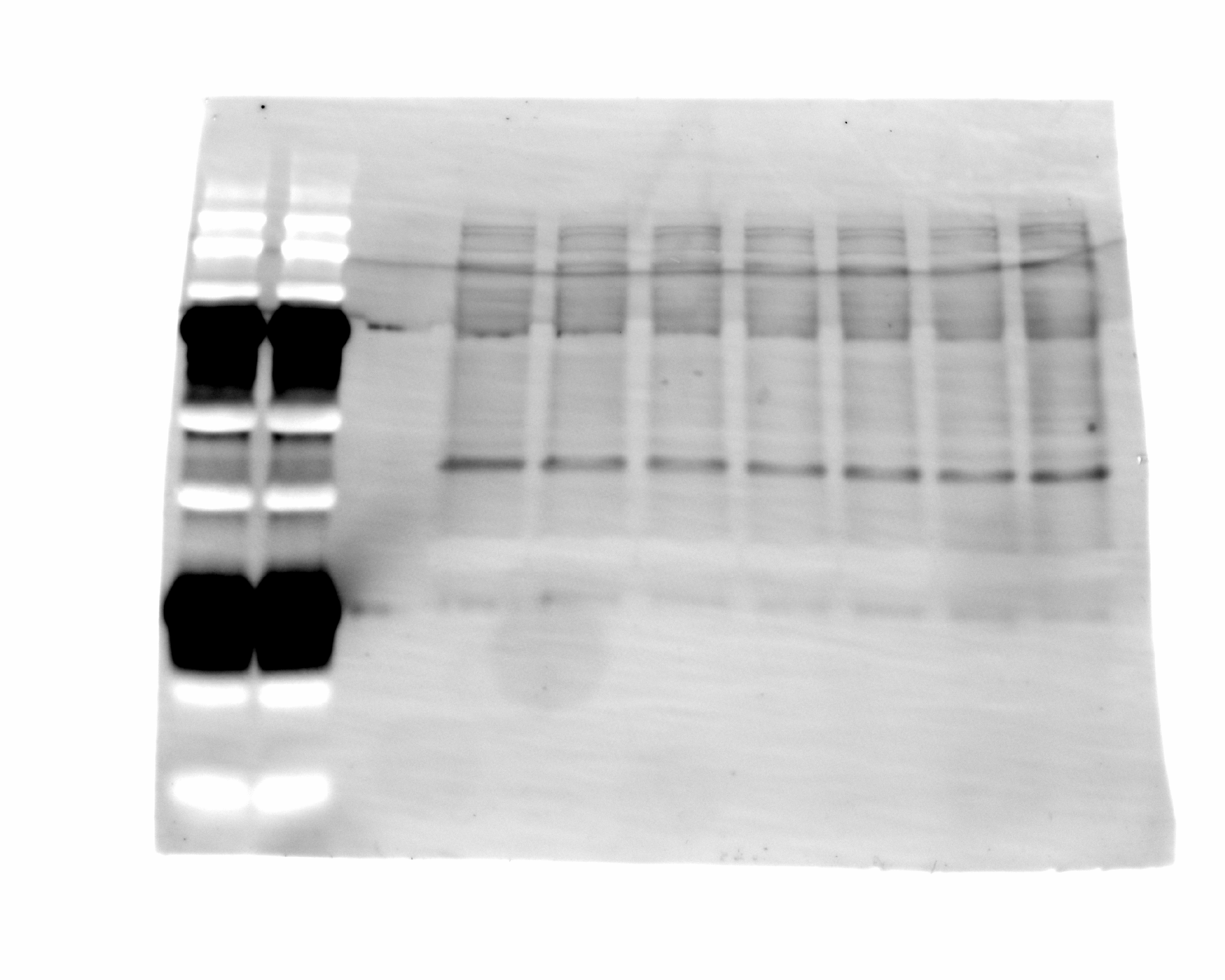

Supplement: Figure 5—source data 3. [file elife-108254-fig5-data3.zip › Figure 5 - source data 1/5h_2_SidCSdcA Rep3 INSOL stainfree .tif]

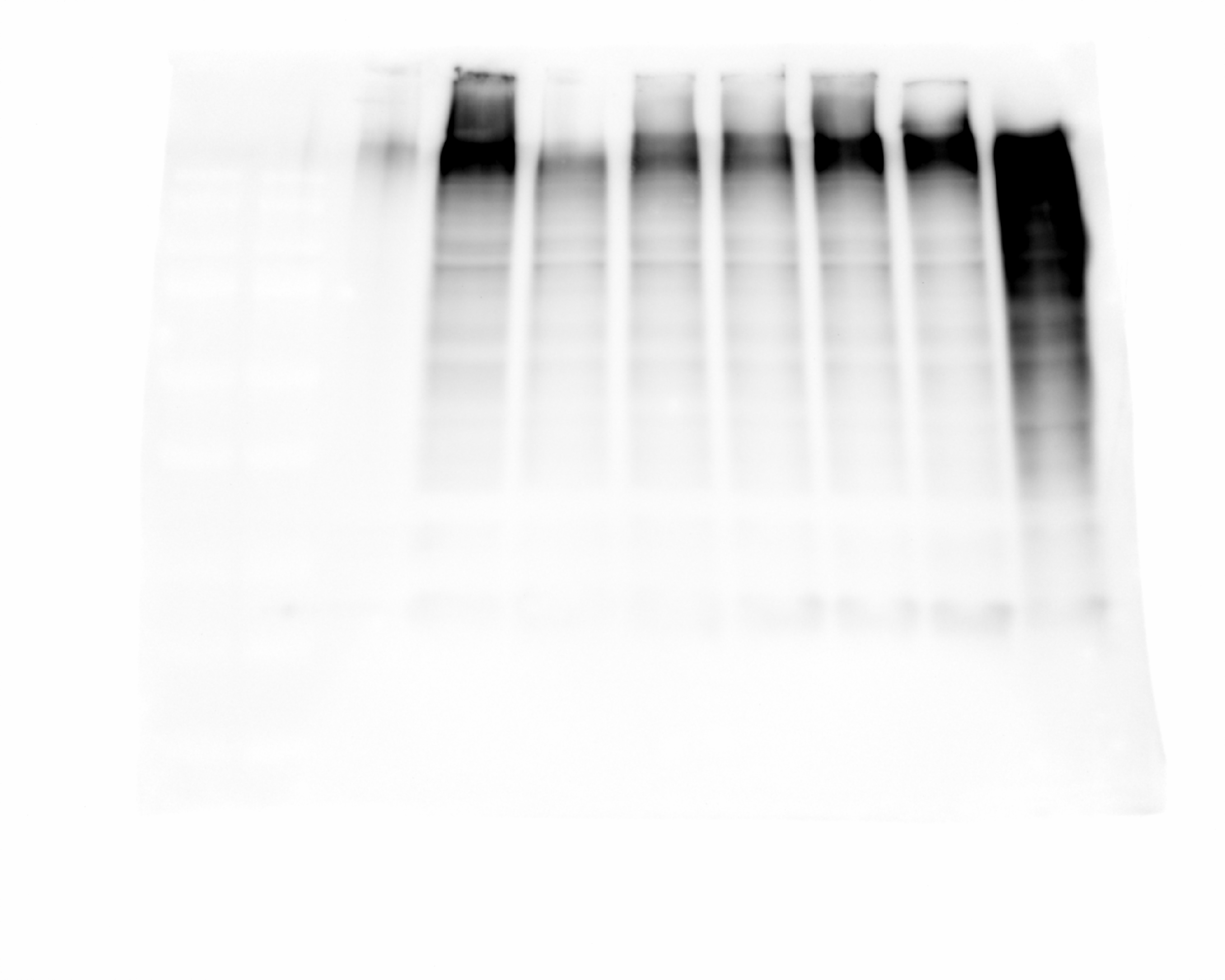

Supplement: Figure 5—source data 3. [file elife-108254-fig5-data3.zip › Figure 5 - source data 1/5h_1_SidCSdcA Rep3 INSOL P4D1.tif]

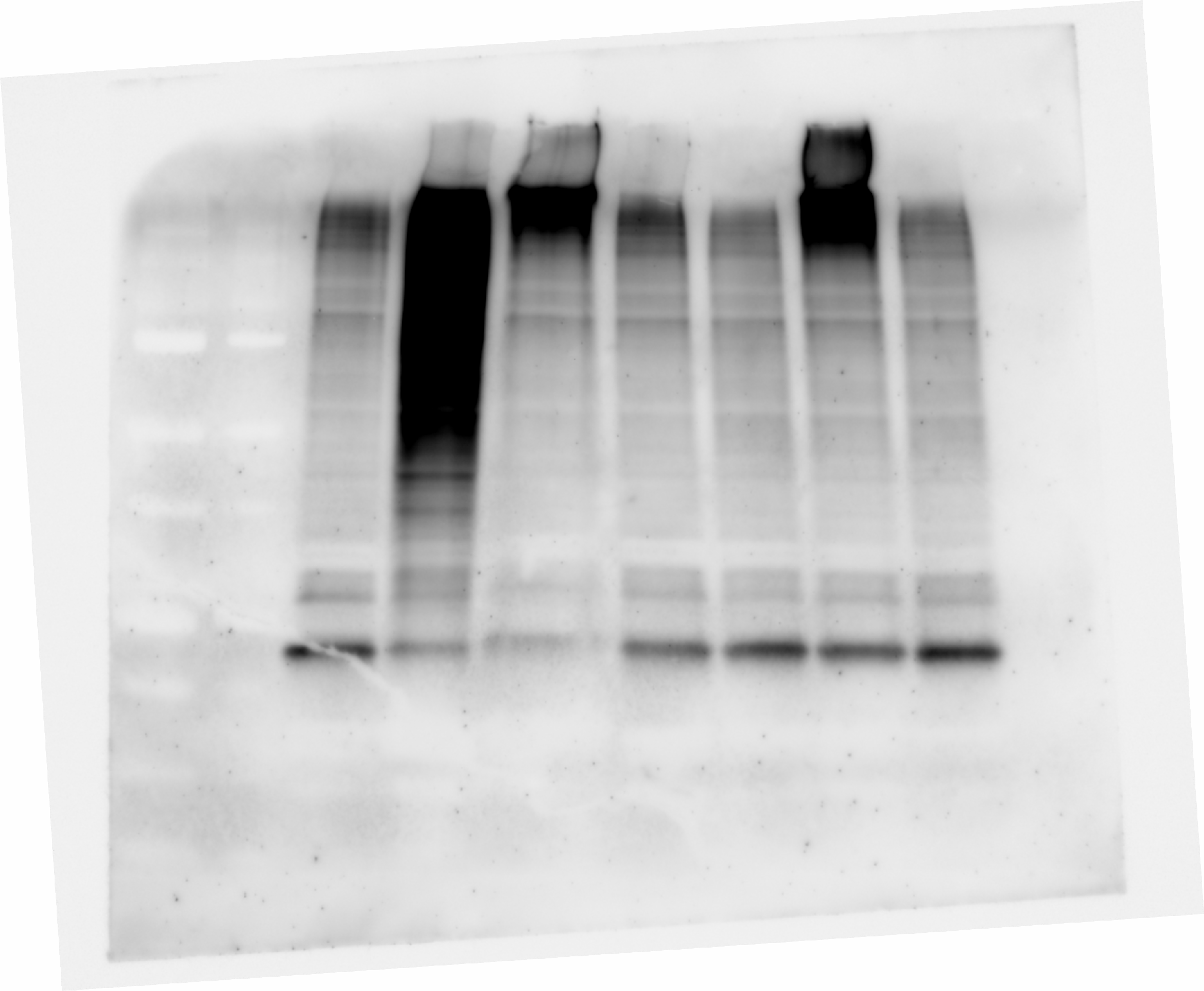

Supplement: Figure 5—source data 3. [file elife-108254-fig5-data3.zip › Figure 5 - source data 1/5e_2_520241007_MET071_InSol_P4D1_68s.tif]

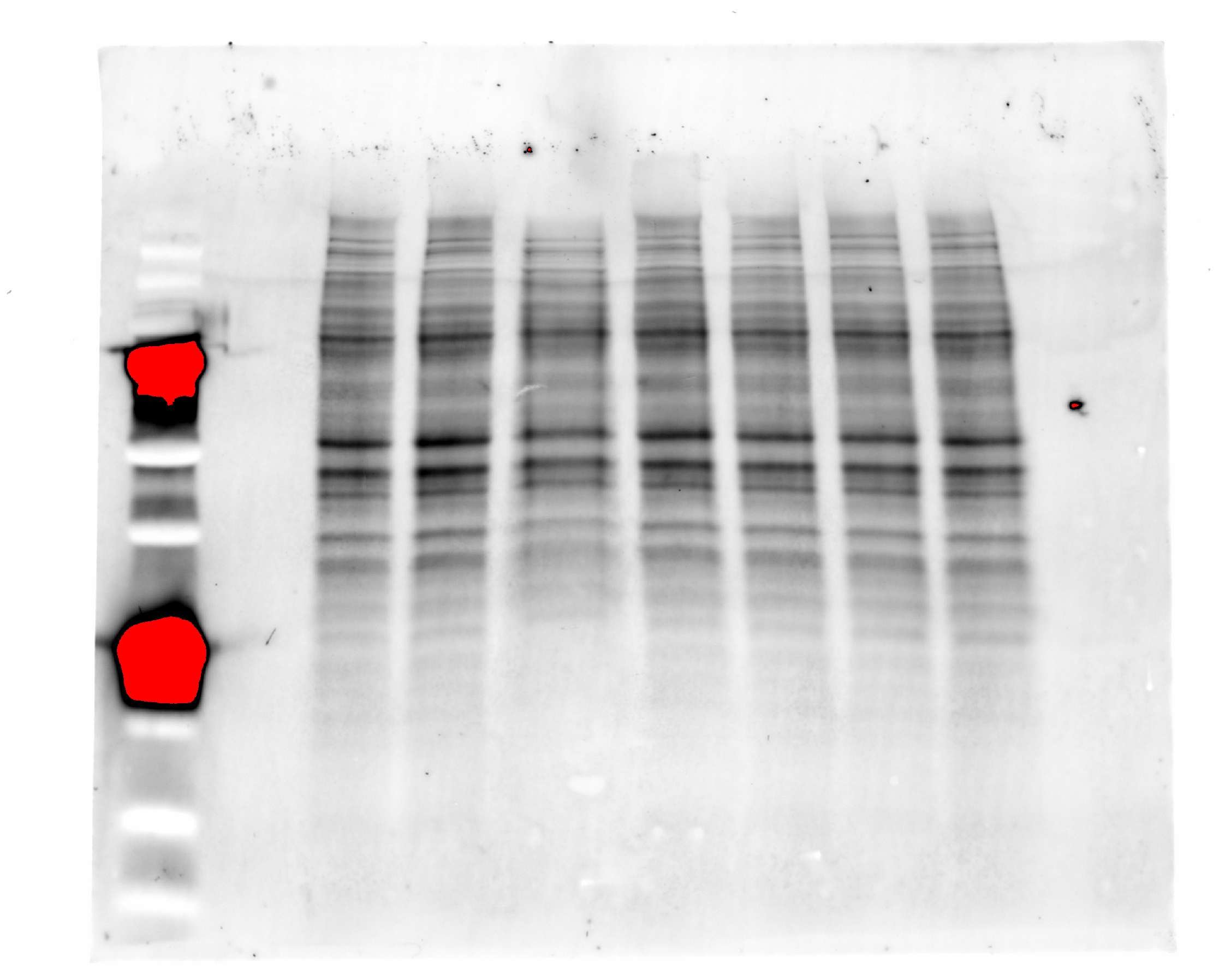

Supplement: Figure 5—source data 3. [file elife-108254-fig5-data3.zip › Figure 5 - source data 1/5d_2_20241007_MET071_Sol_StainFree.tif]

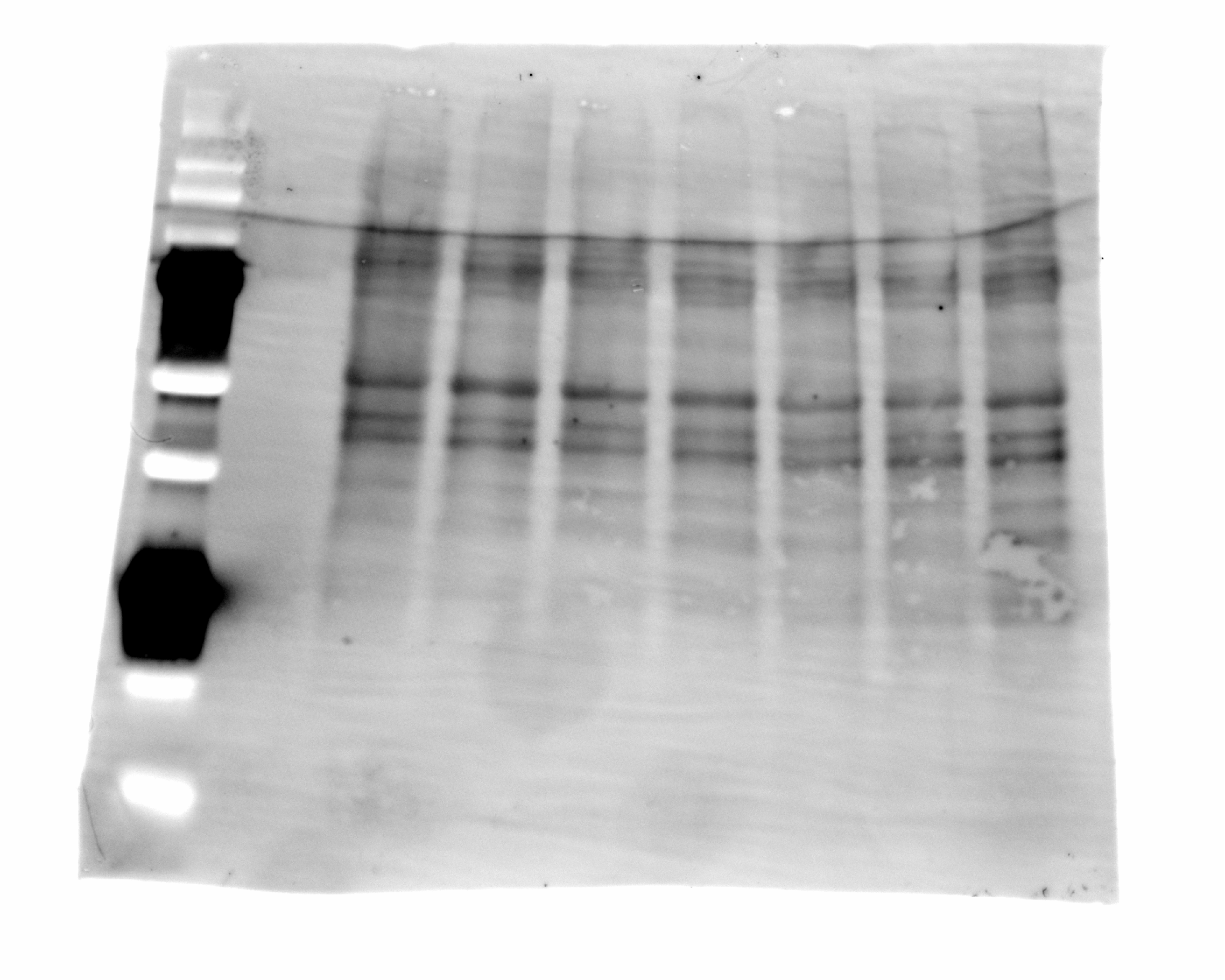

Supplement: Figure 5—source data 3. [file elife-108254-fig5-data3.zip › Figure 5 - source data 1/5g_2_SidCSdcA Rep3 SOLUBLE stainfree .tif]

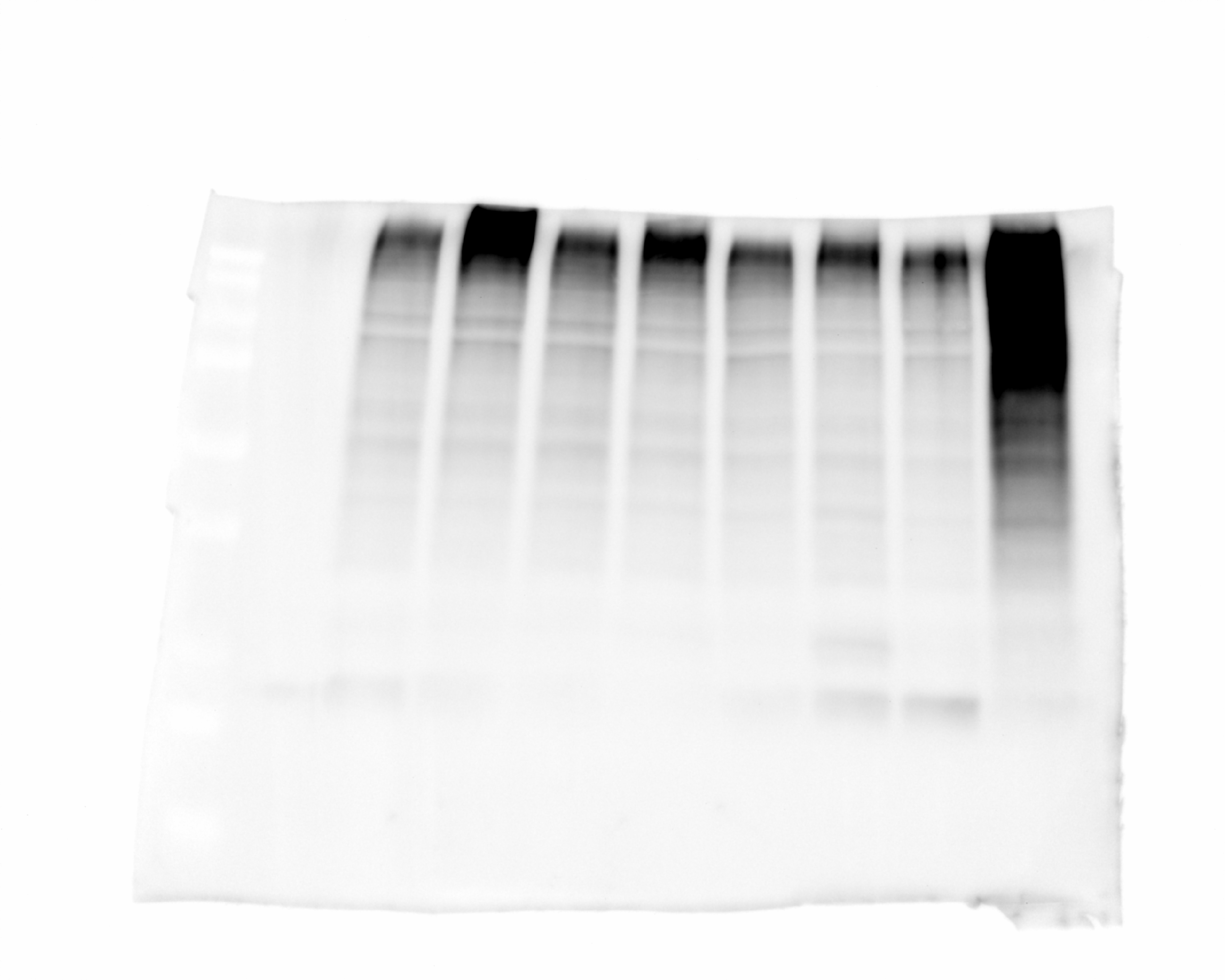

Supplement: Figure 6—source data 2. [file elife-108254-fig6-data2.zip › Figure 6 - source data 1/6d_1_Rep2_P4D1_insol_600s.tif]

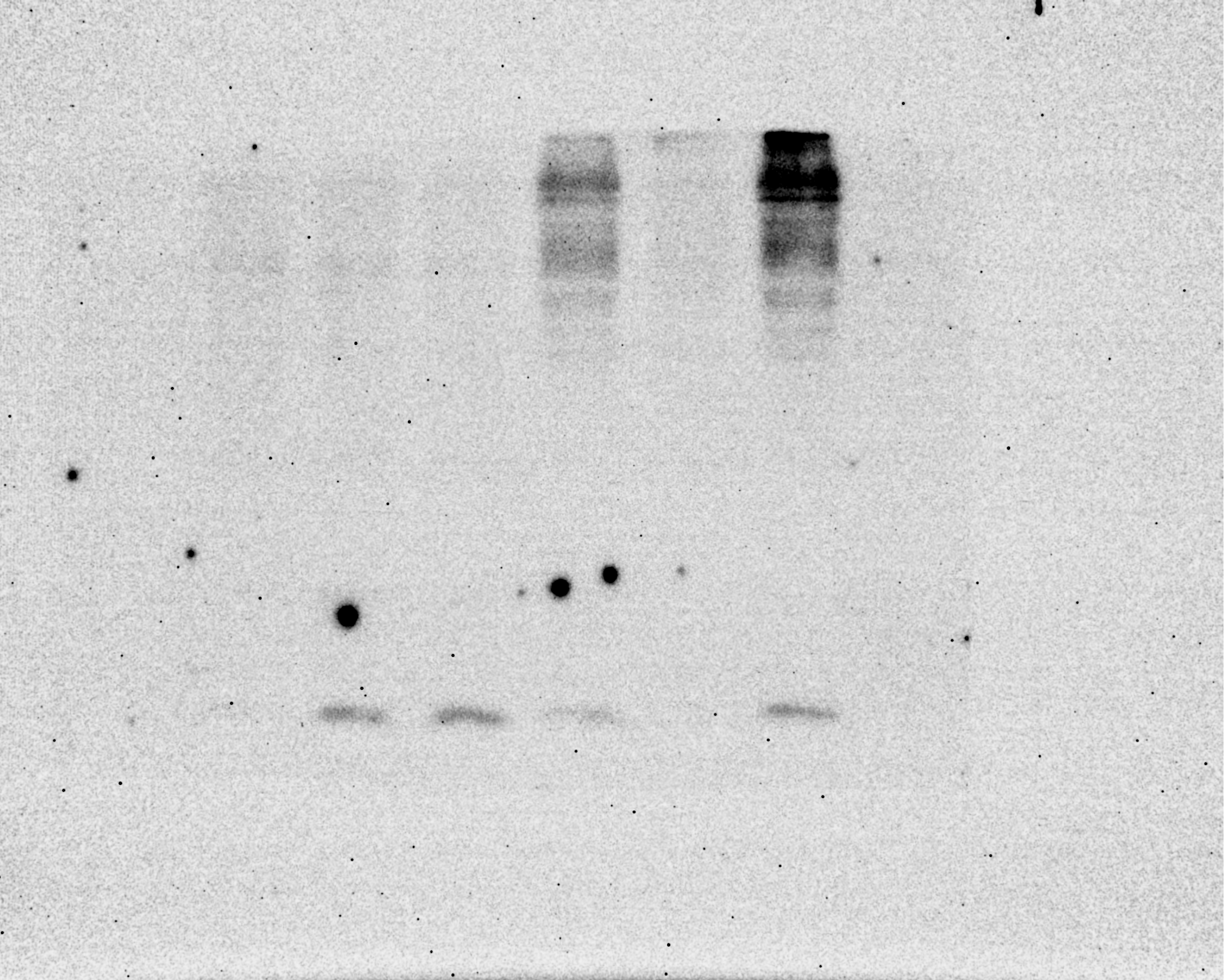

Supplement: Figure 6—source data 2. [file elife-108254-fig6-data2.zip › Figure 6 - source data 1/6s1a_1_HA_600s_chemi.tif]

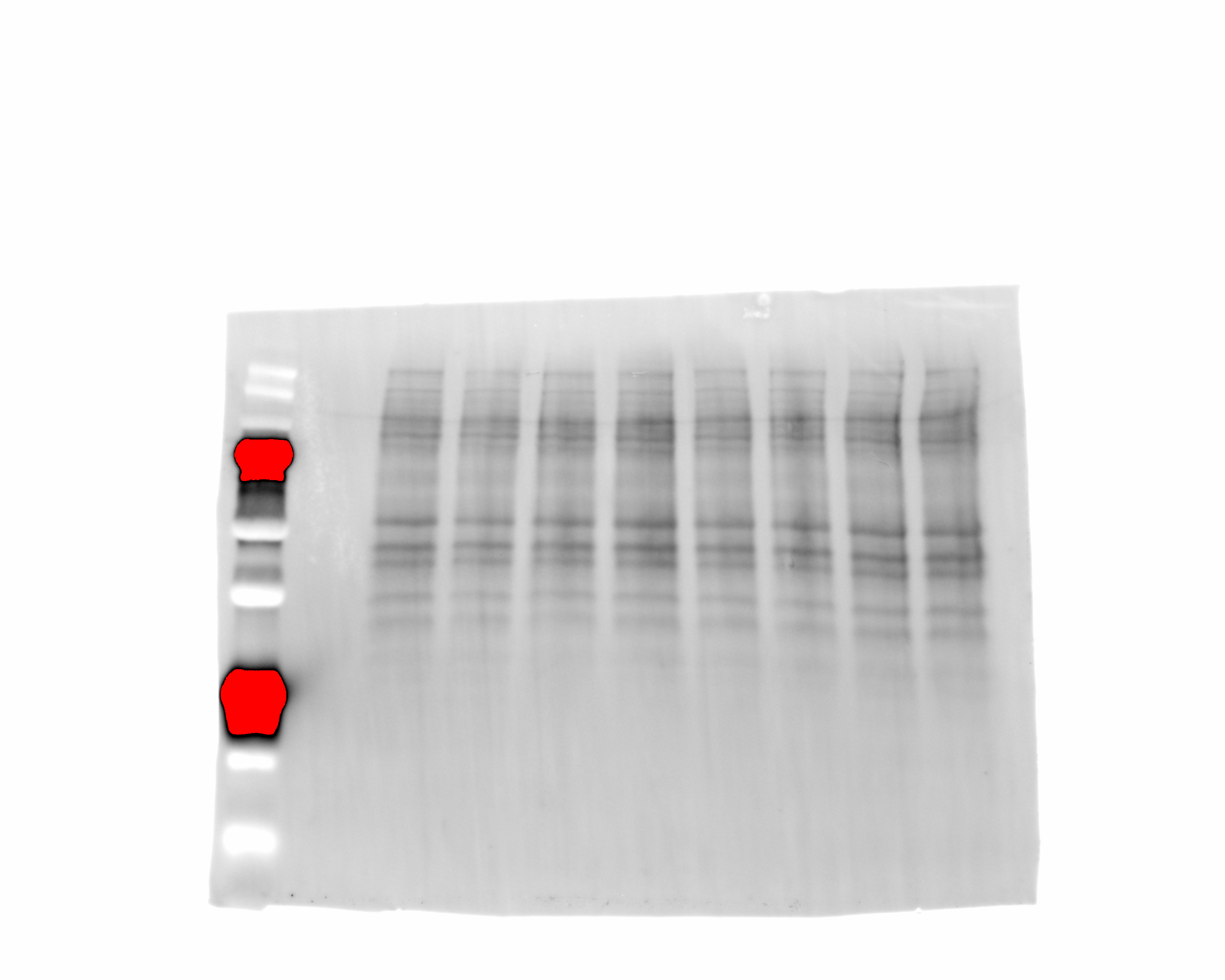

Supplement: Figure 6—source data 2. [file elife-108254-fig6-data2.zip › Figure 6 - source data 1/6c_2_Time Course Rep2 Soluble stainfree.tif]

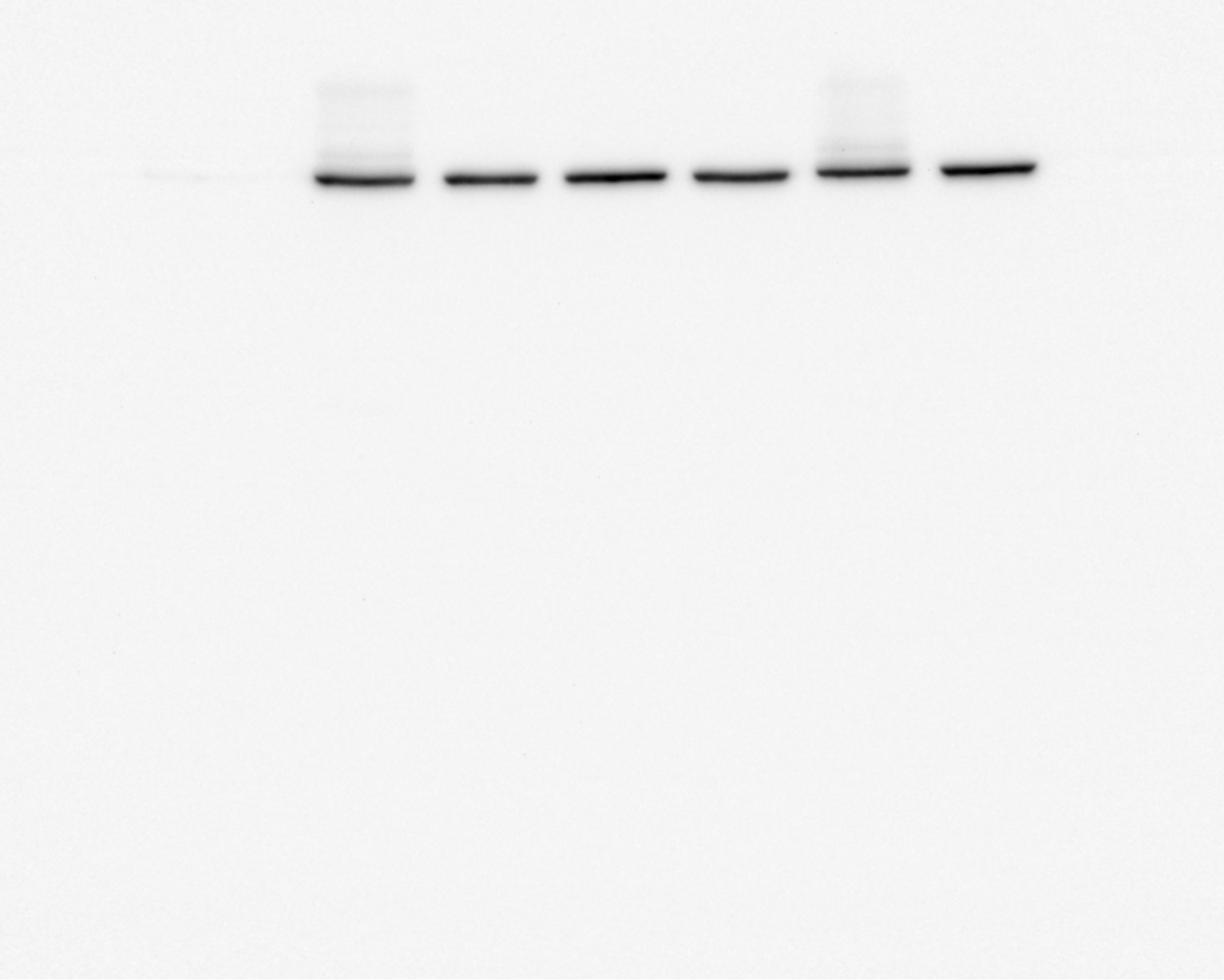

Supplement: Figure 6—source data 2. [file elife-108254-fig6-data2.zip › Figure 6 - source data 1/6s1a_2_Hsp70 chemi DupA expt.tif]

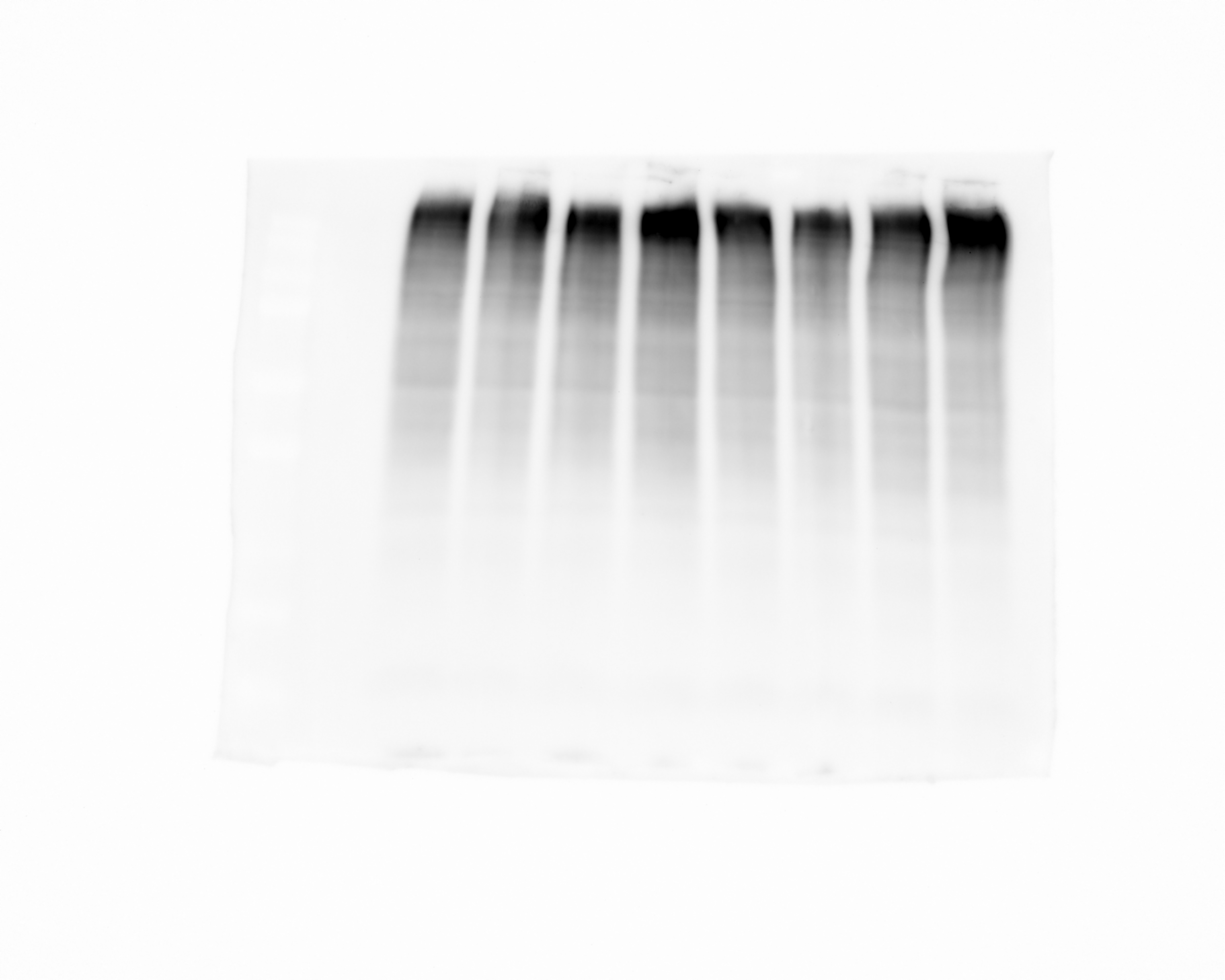

Supplement: Figure 6—source data 2. [file elife-108254-fig6-data2.zip › Figure 6 - source data 1/6c_1_Rep2_P4D1_sol_600s.tif]

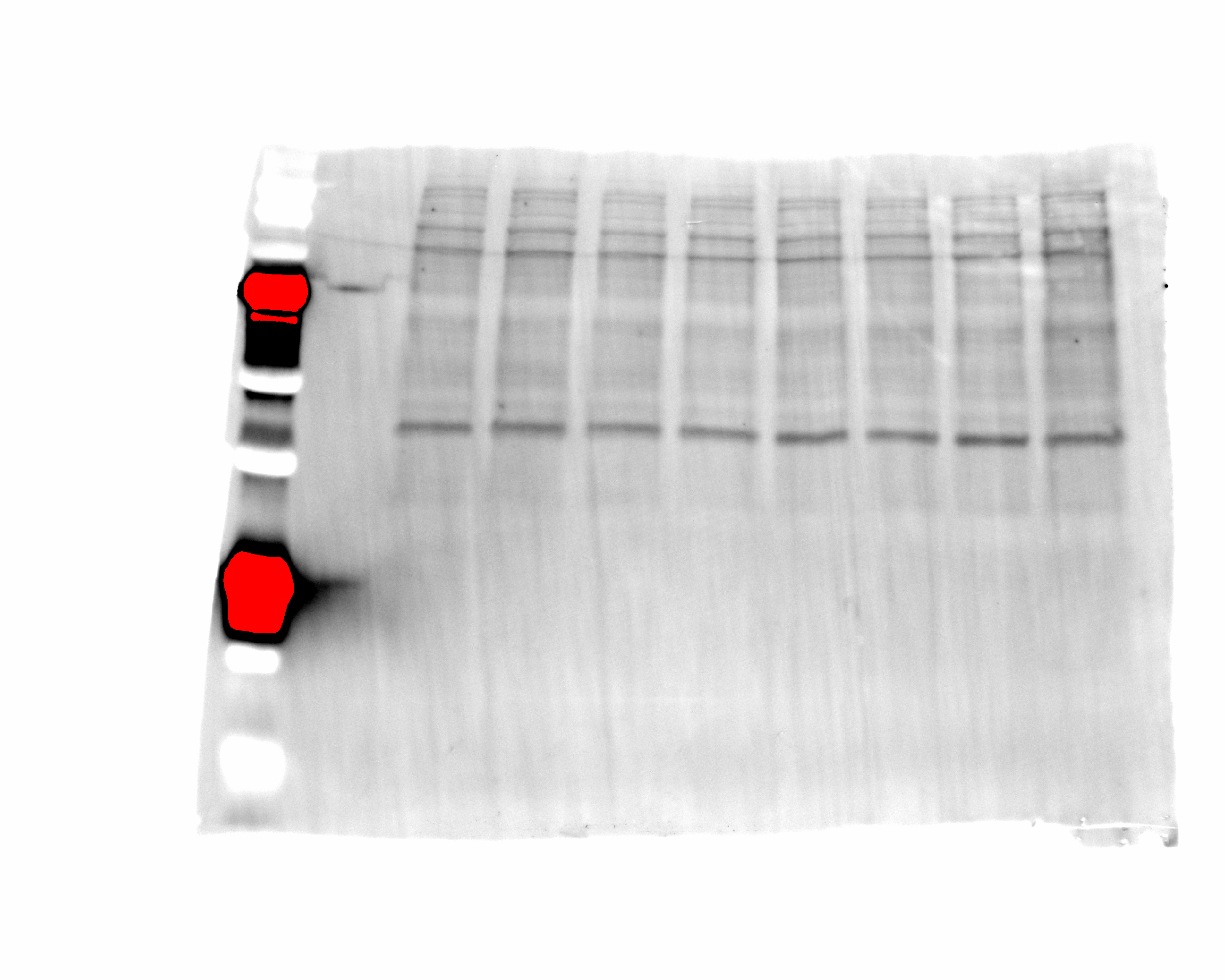

Supplement: Figure 6—source data 2. [file elife-108254-fig6-data2.zip › Figure 6 - source data 1/6d_2_Time Course Rep2 INSOL stainfree.tif]

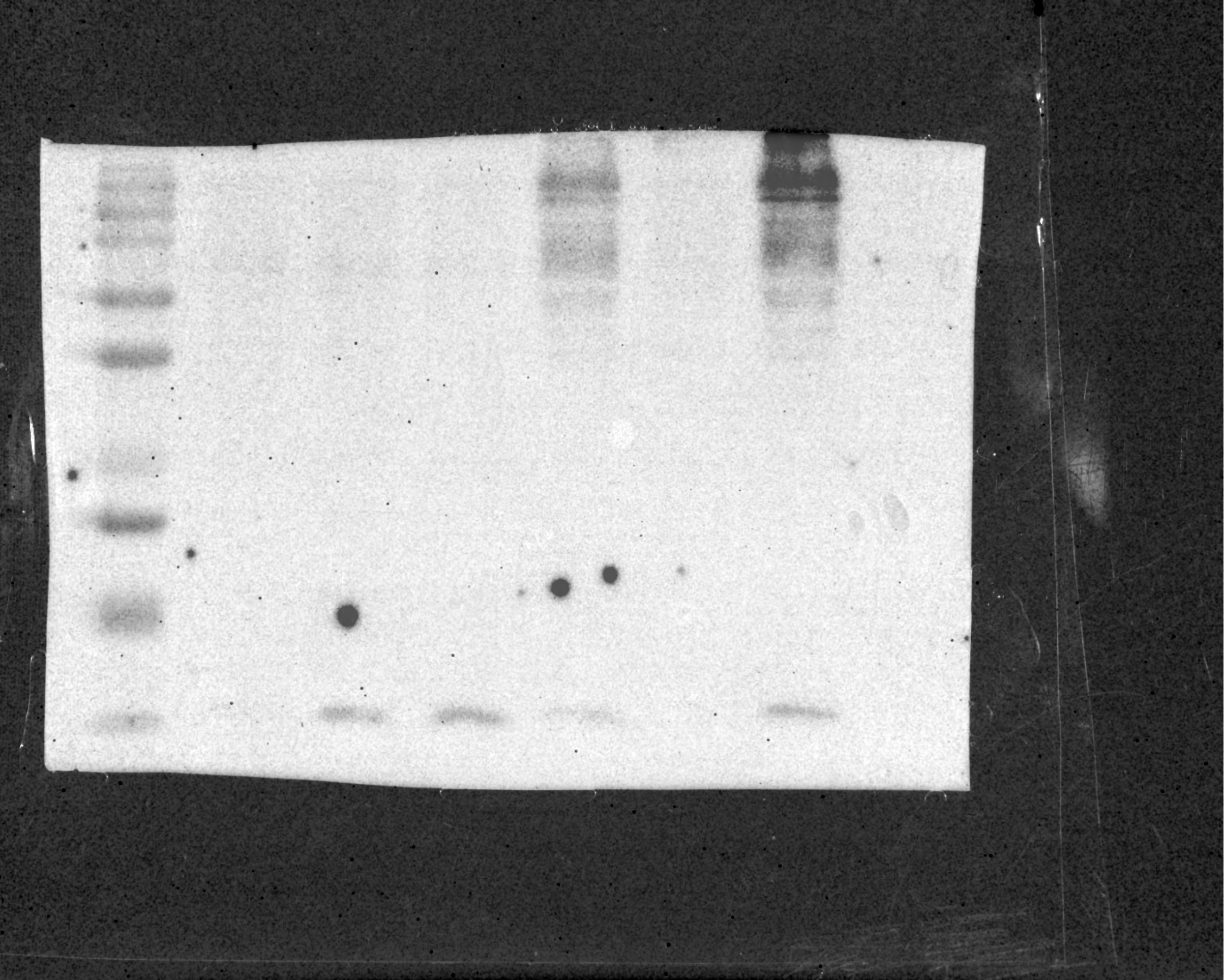

Supplement: Figure 6—figure supplement 1—source data 1. [file elife-108254-fig6-figsupp1-data1.zip › Figure 6, figure supplement 1 - source data 1/6s1A_HA_ladder.tif]

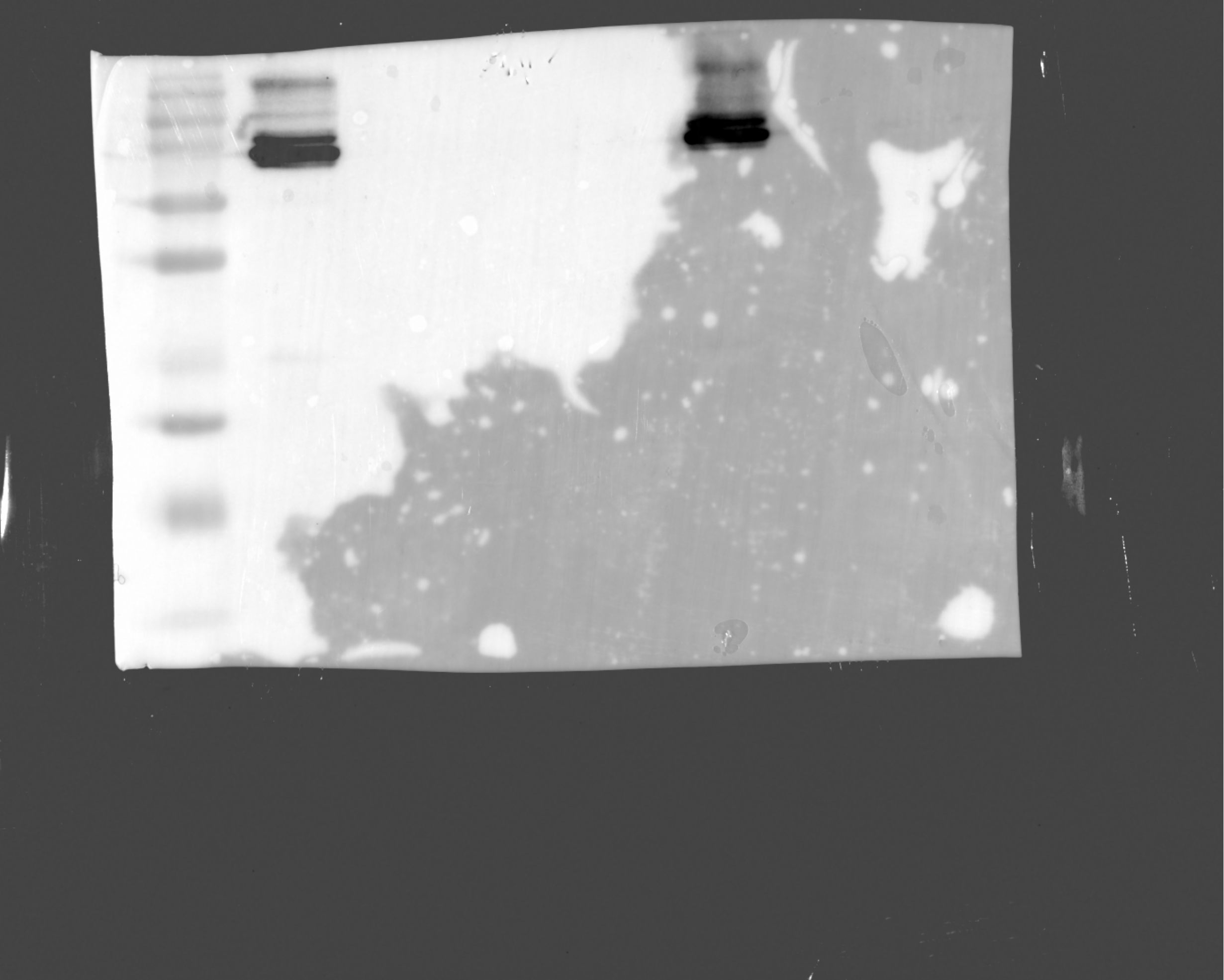

Supplement: Figure 6—figure supplement 1—source data 1. [file elife-108254-fig6-figsupp1-data1.zip › Figure 6, figure supplement 1 - source data 1/6s1A_gfp_ladder.tif]

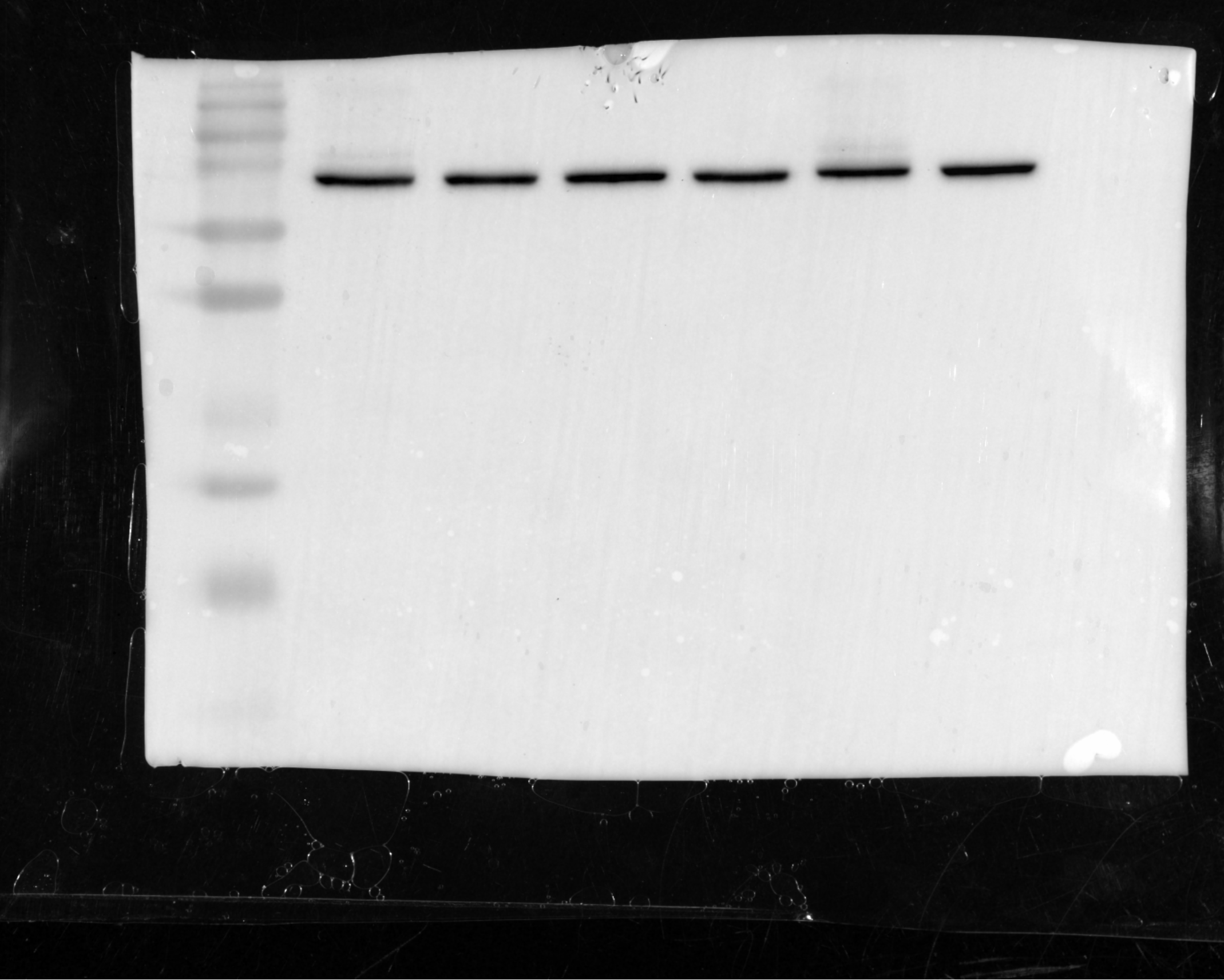

Supplement: Figure 6—figure supplement 1—source data 1. [file elife-108254-fig6-figsupp1-data1.zip › Figure 6, figure supplement 1 - source data 1/6s1A_hsp70_ladder.tif]
