# Supplementary material for: Canonical and phosphoribosyl ubiquitination coordinate to stabilize a proteinaceous structure surrounding the Legionella-containing vacuole
Source: eLife. 2026 Jul 8;14:RP108254. doi: 10.7554/eLife.108254 (PMC13345631; doi:10.7554/eLife.108254)
Supplement: Figure 1—figure supplement 1—source data 2. [file elife-108254-fig1-figsupp1-data2.zip › Figure 1, figure supplement 1 - source data 2/Figure 1 source data labeled.pdf]

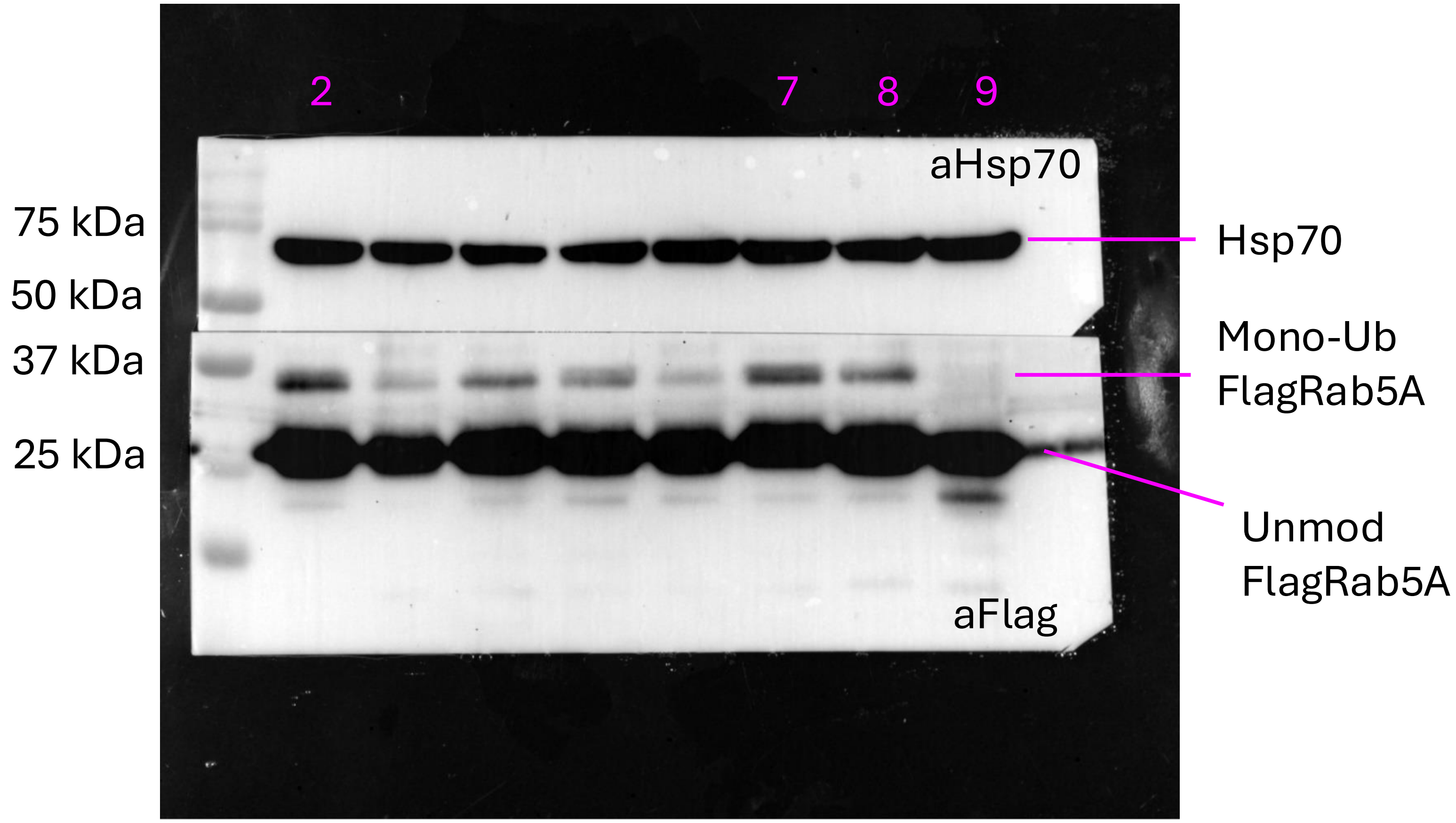

Source image for Figure 1, supplement 1c. Colorimetric image of BioRad Precision Plus Dual-Stained ladder (Lane 1) merged with chemiluminescence image. All samples are whole cell lysates from HEK FcGR cells transiently transfected with the indicated construct and infected with WT *Legionella pneumophila* for 5 hours. Note that the second half of the experiment was not discussed in the manuscript and therefore is not labeled here for the sake of clarity.

Lane 2: Flag-Rab5A WT
Lane 7: Flag-Rab5A Q79L
Lane 8: Flag-Rab5A S34N
Lane 9: Flag-Rab5A 1-211
