# Supplementary material for: Canonical and phosphoribosyl ubiquitination coordinate to stabilize a proteinaceous structure surrounding the Legionella-containing vacuole
Source: eLife. 2026 Jul 8;14:RP108254. doi: 10.7554/eLife.108254 (PMC13345631; doi:10.7554/eLife.108254)
Supplement: Figure 2—source data 3. [file elife-108254-fig2-data3.zip › Figure 2 - source data 2/Figure 2 source data 2.pdf]

aHA (HA-ubiquitin dGG)

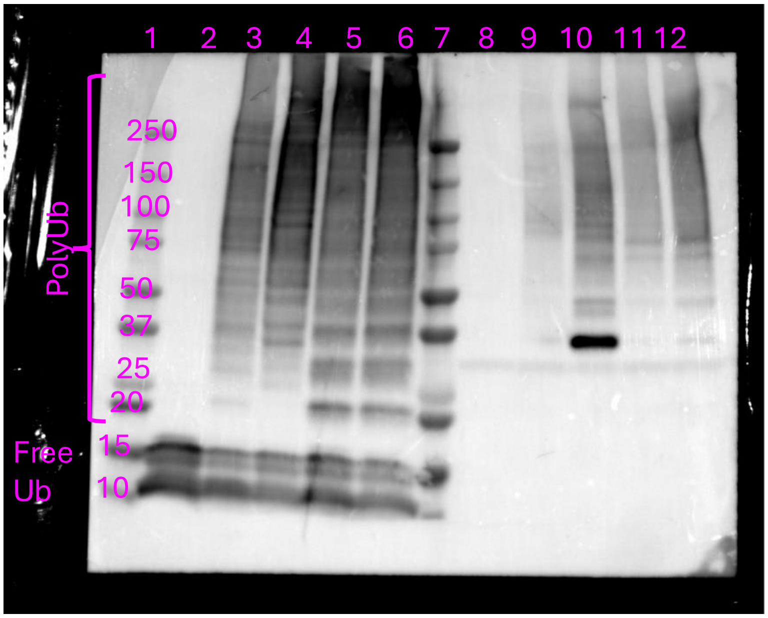

aMyc (Myc-tagged effectors)

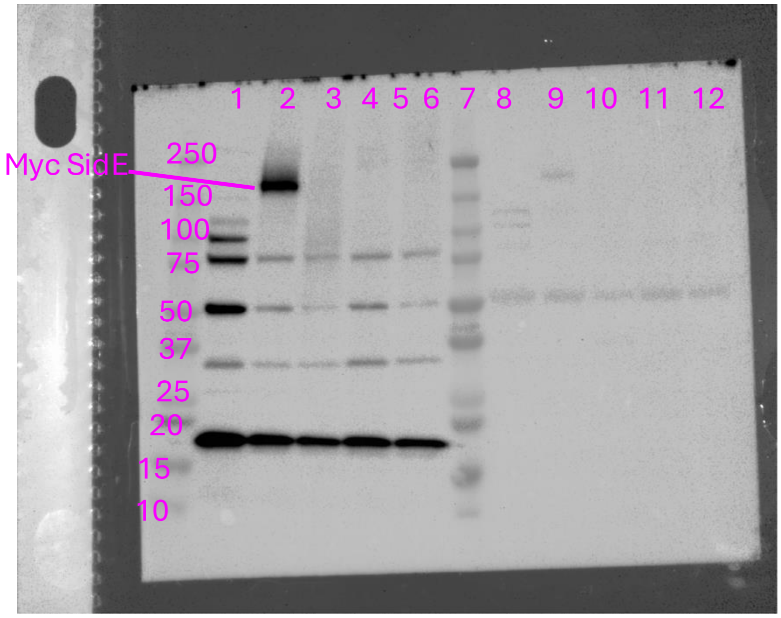

aFlag (Flag-Rab5A)

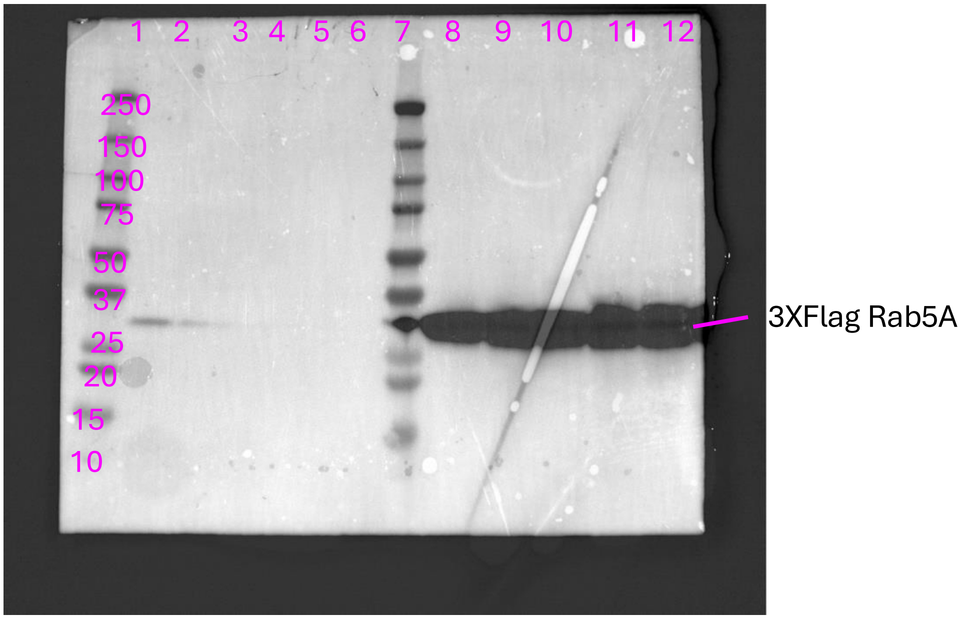

Lanes:

1. Ladder
2. Vector (input)
3. Myc SidE (input)
4. Myc SdeA (input)
5. Myc SdeB (input)
6. Myc SdeC (input)
7. Ladder
8. Vector (Flag IP)
9. Myc SidE (Flag IP)
10. Myc SdeA (Flag IP)
11. Myc SdeB (Flag IP)
12. Myc SdeC (Flag IP)

Source data for Figure 2, panel C. HEK293T FcGR cells transfected with Flag-Rab5A, HA-ubiquitin dGG, and the indicated *Legionella* effector (or vector control) were lysed and subjected to Flag IP to isolate Flag-tagged Rab5. All blots are merged chemiluminescence and colorimetric images of the Dual Stained Precision Plus ladder (BioRad), ladder label units are kDa.

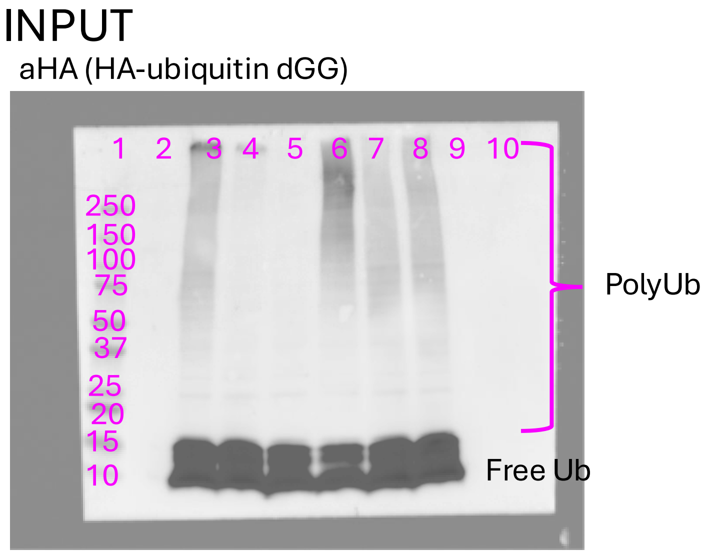

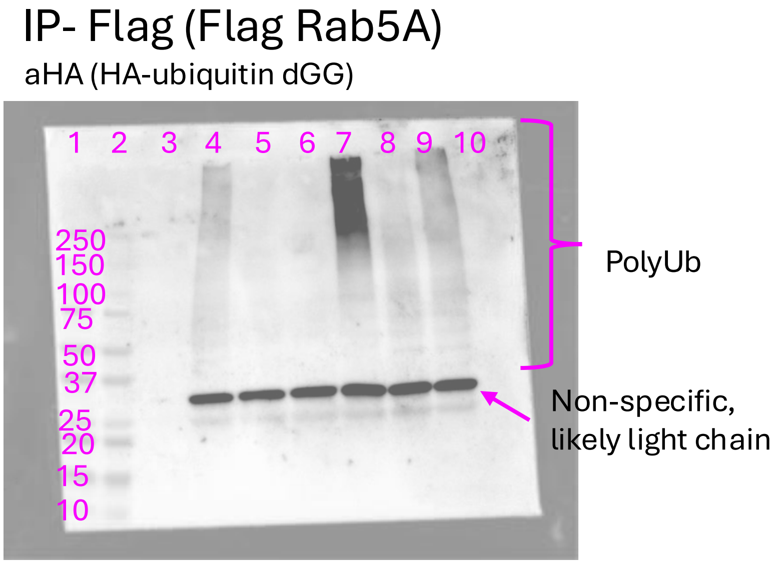

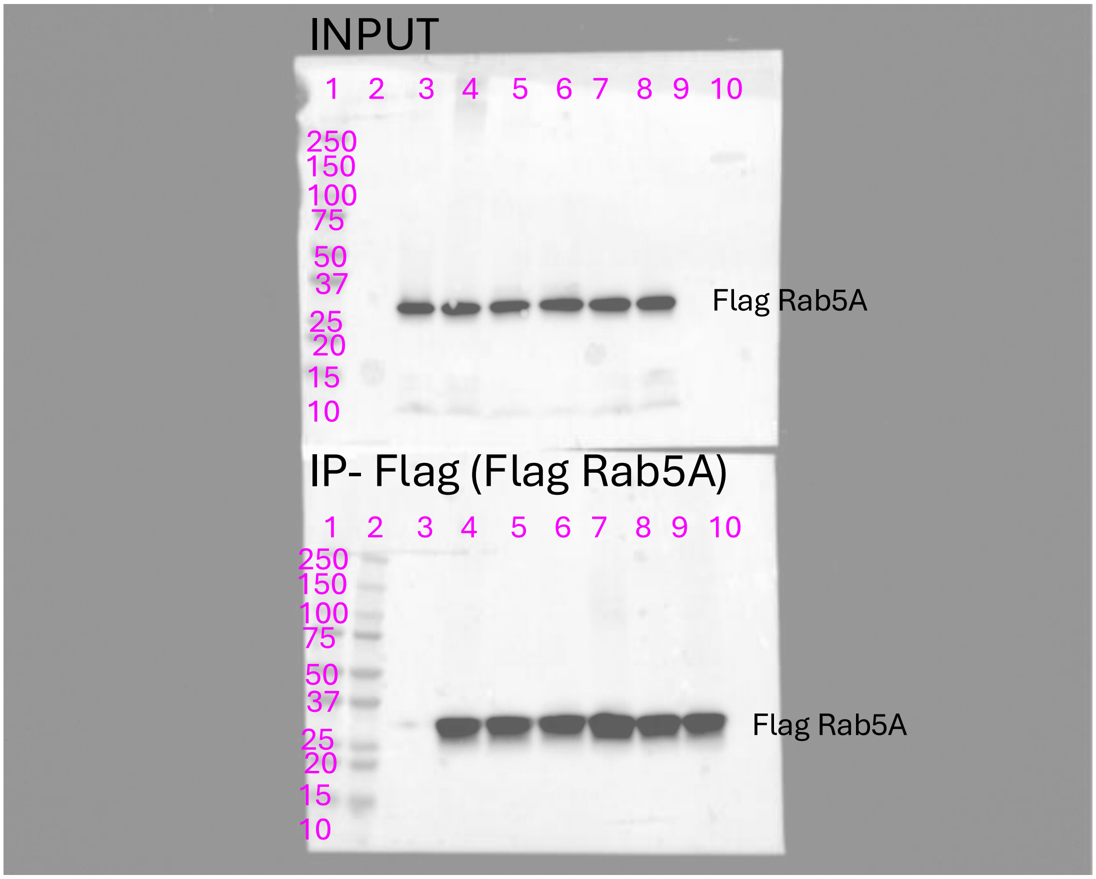

Lanes - input

1. Ladder
2. Buffer
3. WT *L.p.*
4. *dotA L.p.*
5. dSidE family
6. dSidE family + pSdeB

Lanes – Flag IP

1. Ladder
2. Ladder
3. Buffer
4. WT *L.p.*
5. *dotA L.p.*
6. dSidE family
7. dSidE family + pSdeB

Source data for Figure 2, panel D. HEK293T FcGR cells transfected with Flag-Rab5A, HA-ubiquitin dGG, and infected for 1 hr with the indicated *Legionella* strain were lysed and subjected to Flag IP to isolate Flag-tagged Rab5. All blots are merged chemiluminescence and colorimetric images of the Dual Stained Precision Plus ladder (BioRad), ladder label units are kDa. Note that all remaining lanes not included in lane labels are samples from cells infected with additional *Legionella* strains, but as this data is not discussed in the paper, these lanes are left unlabeled for clarity.

aHA (HA-ubiquitin dGG)

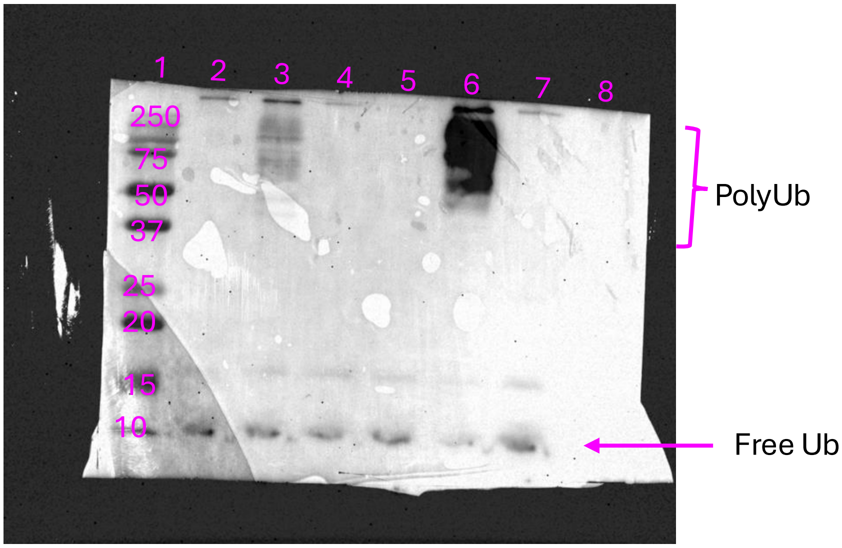

Lanes:

1. Ladder
2. Uninfected
3. WT *L.p.*
4. *dotA L.p.*
5. dSidE family
6. dSidE family + pSdeB
7. dSidE family + pSdeB EE/AA
8. Buffer

aHsp70

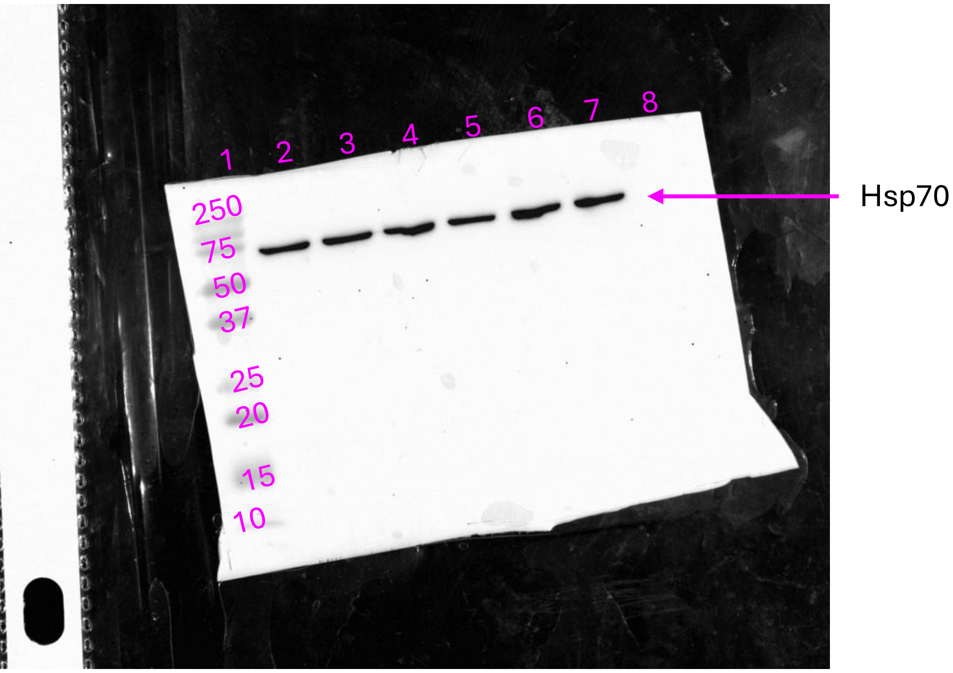

Source data for Figure 2, supplement 1. Western blot analysis of HA-Ub DGG conjugation in whole cell lysates prepared from HEK293T FcgR cells infected with the indicated *L.p.* strain for 1 hour. All blots are merged chemiluminescence and colorimetric images of the Dual Stained Precision Plus ladder (BioRad), ladder label units are kDa.
