# Supplementary material for: Canonical and phosphoribosyl ubiquitination coordinate to stabilize a proteinaceous structure surrounding the Legionella-containing vacuole
Source: eLife. 2026 Jul 8;14:RP108254. doi: 10.7554/eLife.108254 (PMC13345631; doi:10.7554/eLife.108254)
Supplement: Figure 2—figure supplement 1—source data 2. [file elife-108254-fig2-figsupp1-data2.zip › Figure 2, figure supplement 1 - source data 2/Figure 2, figure supplement 1 source data 2.pdf]

aHA (HA-ubiquitin dGG)

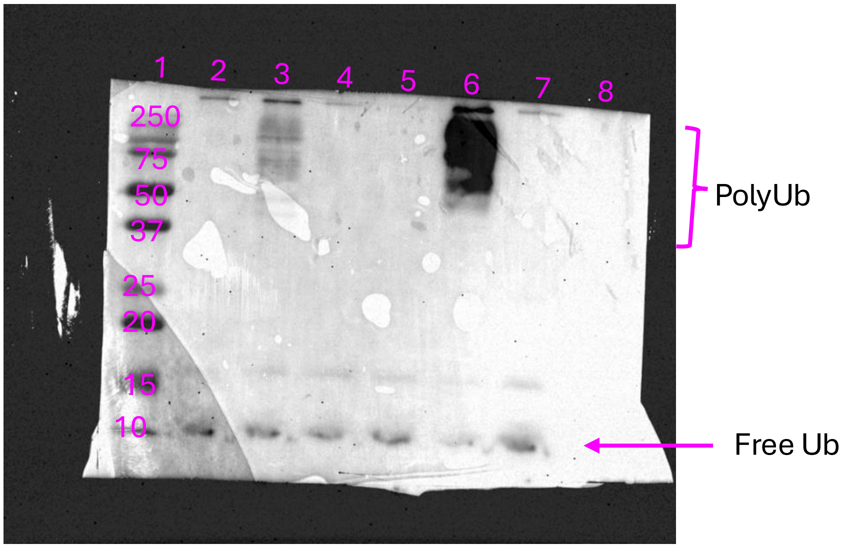

Lanes:

1. Ladder
2. Uninfected
3. WT *L.p.*
4. *dotA L.p.*
5. dSidE family
6. dSidE family + pSdeB
7. dSidE family + pSdeB EE/AA
8. Buffer

aHsp70

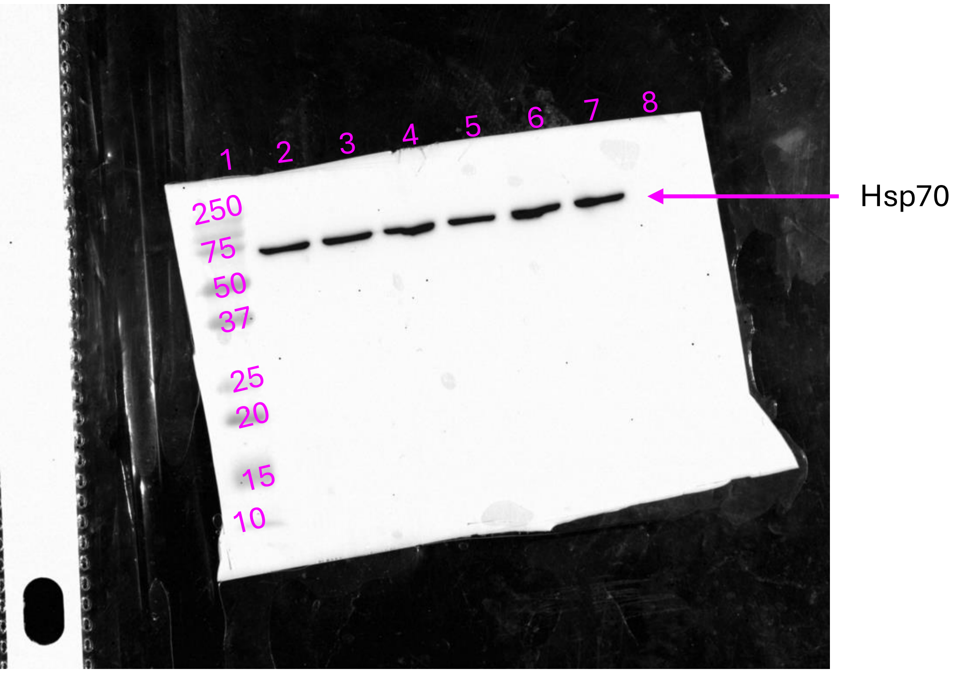

Source data for Figure 2, supplement 1. Western blot analysis of HA-Ub DGG conjugation in whole cell lysates prepared from HEK293T FcgR cells infected with the indicated *L.p.* strain for 1 hour. All blots are merged chemiluminescence and colorimetric images of the Dual Stained Precision Plus ladder (BioRad), ladder label units are kDa.
