# Supplementary material for: Canonical and phosphoribosyl ubiquitination coordinate to stabilize a proteinaceous structure surrounding the Legionella-containing vacuole
Source: eLife. 2026 Jul 8;14:RP108254. doi: 10.7554/eLife.108254 (PMC13345631; doi:10.7554/eLife.108254)
Supplement: Figure 6—source data 3. [file elife-108254-fig6-data3.zip › Figure 6 - source data 2/Figure 6 source data labeled.pdf]

SOLUBLE FRACTION

aUbiquitin (P4D1)

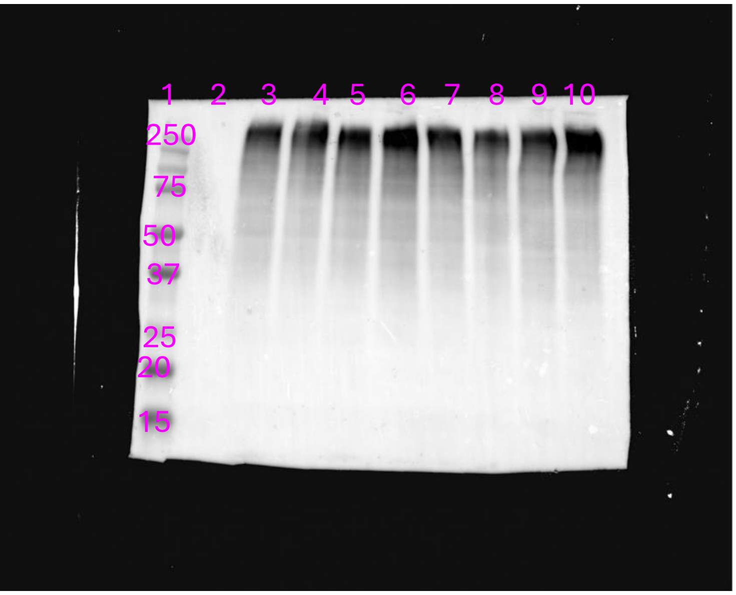

Stain Free

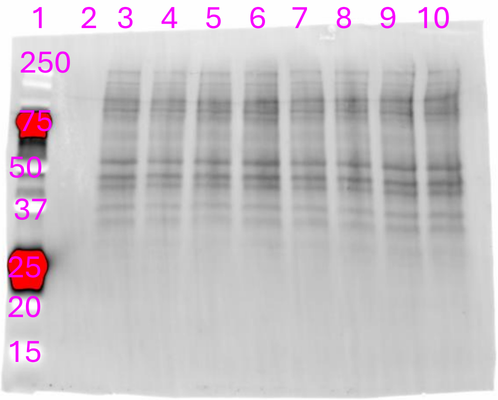

Lanes

1. Ladder
2. Buffer
3. Uninfected
4. WT *L.p.,* 1HR
5. *dotA L.p.,* 1HR
6. WT *L.p.*, 4HR
7. *dotA L.p.,* 4HR
8. WT *L.p.*, 8HR
9. *dotA L.p.*, 8HR
10. Heat shock

INSOLUBLE FRACTION

aUbiquitin (P4D1)

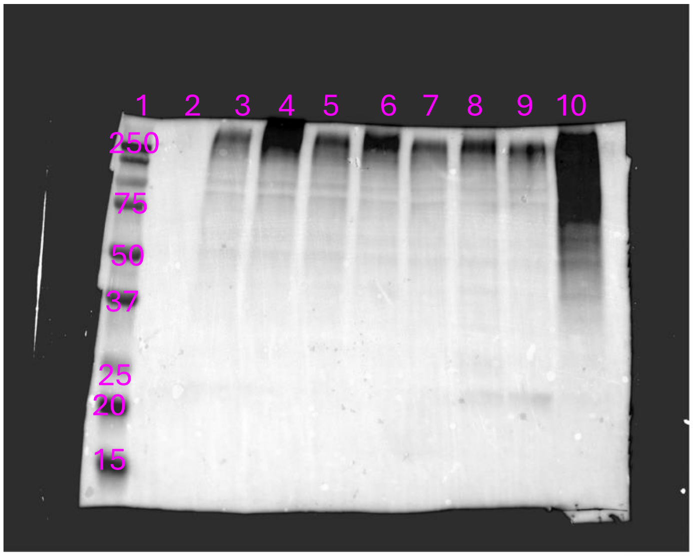

Stain Free

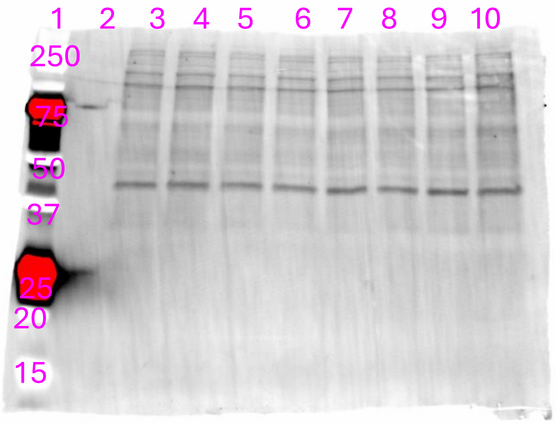

Lanes

1. Ladder
2. Buffer
3. Uninfected
4. WT *L.p.,* 1HR
5. *dotA L.p.,* 1HR
6. WT *L.p.*, 4HR
7. *dotA L.p.,* 4HR
8. WT *L.p.*, 8HR
9. *dotA L.p.*, 8HR
10. Heat shock

Source data for Figure 6, C-D. Immunoblot analysis of total ubiquitin in the (C) soluble or (D) insoluble fraction of cells infected with the indicated *L.p.* strain for the indicated duration, heat shocked for 1 HR at 42C, or left untreated, with total protein (StainFree, BioRad) shown as the loading control. Ubiquitin blots are merged chemiluminescence and colorimetric images of the Dual Stained Precision Plus ladder (BioRad), ladder label units are kDa. Stain free blot size ladder labeling is based on ladder background in StainFree image.
