# Supplementary material for: Canonical and phosphoribosyl ubiquitination coordinate to stabilize a proteinaceous structure surrounding the Legionella-containing vacuole
Source: eLife. 2026 Jul 8;14:RP108254. doi: 10.7554/eLife.108254 (PMC13345631; doi:10.7554/eLife.108254)
Supplement: Figure 6—figure supplement 1—source data 2. [file elife-108254-fig6-figsupp1-data2.zip › Figure 6, figure supplement 1 - source data 2/Figure 6 figure supplement 1 source data labeled.pdf]

aHA (HA-ubiquitin dGG)

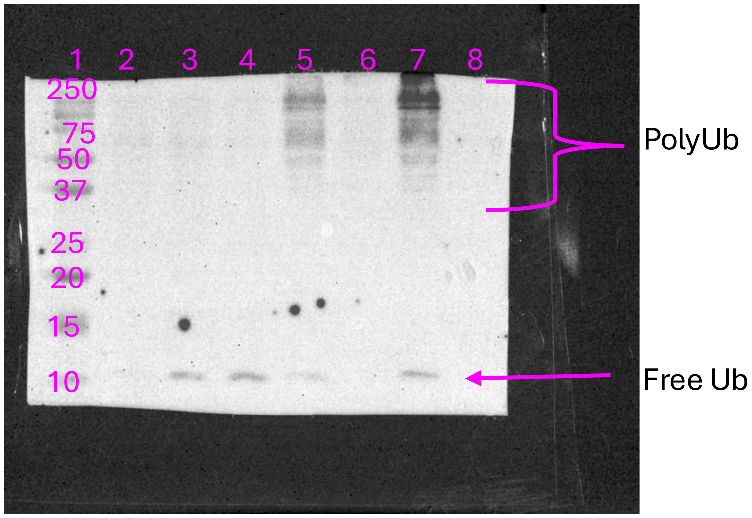

aHsp70

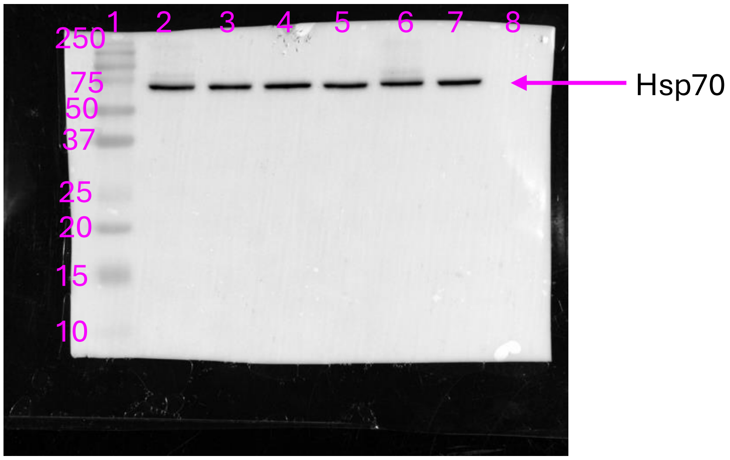

aGFP (GFP-DupA)

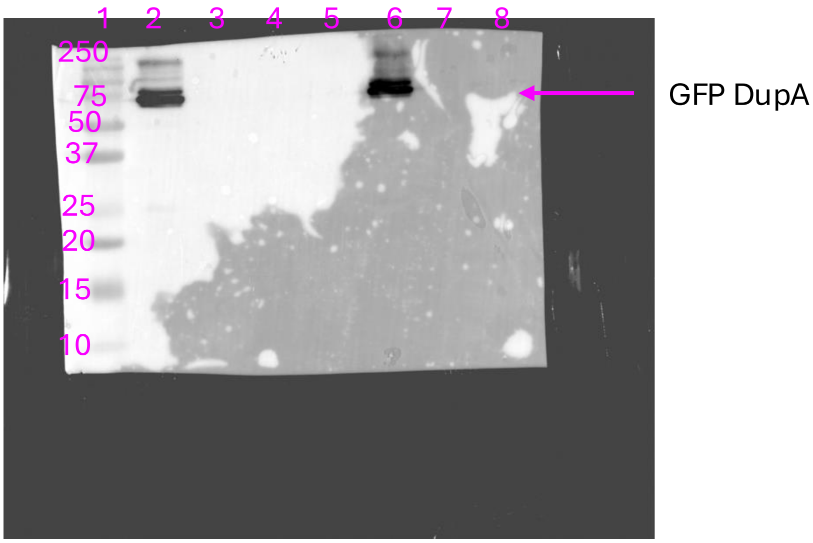

Lanes

1. Ladder
2. GFP DupA, uninfected
3. LotC, uninfected
4. Vector, uninfected
5. LotC, WT *L.p.*
6. GFP DupA, WT *L.p.*
7. Vector, WT *L.p.*

Source data for Figure 6, supplement 1 A. Western blot analysis of lysates from cells transfected with HA-ubiquitin DGG and the indicated construct (LotC is a canonical ubiquitin ligase effector), and either left uninfected or infected with *L.p.* WT for 1 HR. All blots are merged chemiluminescence and colorimetric images of the Dual Stained Precision Plus ladder (BioRad), ladder label units are kDa.
