# Supplementary material for: Canonical and phosphoribosyl ubiquitination coordinate to stabilize a proteinaceous structure surrounding the Legionella-containing vacuole
Source: eLife. 2026 Jul 8;14:RP108254. doi: 10.7554/eLife.108254 (PMC13345631; doi:10.7554/eLife.108254)
Supplement: Peer review history

# Editor report: eLife Assessment

**DOI:** 10.7554/eLife.108254.3.sa0

**Authors:** Dario S Zamboni

**Affiliations:** Universidade de Sao Paulo Brazil

**Keywords:** Compelling, Important

The authors describe the **important** finding that the *Legionella*-containing vacuole is surrounded by a dense ubiquitin ‘cloud’ that is highly resistant to detergent extraction. The study provides **compelling** evidence that this structure is generated through a combination of canonical ubiquitination mediated by the SidC *Legionella* ligase and phosphoribosyl-ubiquitination mediated by the SidE family of *Legionella* ligases. These findings provide insight into how *Legionella* remodels the host vacuolar environment through complex ubiquitin modifications.

# Referee report: Reviewer #1 (Public review)

**DOI:** 10.7554/eLife.108254.3.sa1

[Editors' note: this version has been assessed by the Reviewing Editor without further input from the original reviewers. The authors have addressed the reviewers' comments adequately and revised the manuscript accordingly.]

Summary:

In the submitted manuscript, Steinbach et al describe the formation of a detergent-resistant "cloud" around the Legionella-containing vacuole (LCV) that functions as a protective barrier. The authors show that formation of the "cloud" barrier is contingent upon the phosphoribosyl-ubiquitination activity of the SidE/SdeABC effector family, and is temporally regulated, with the assembly and subsequent disassembly of the "cloud" coinciding with replication and vacuolar expansion. The authors postulate a model of "cloud" barrier formation that relies upon a wave of initial ubiquitination by the SidC effector family, after which the SidE/SdeABC family expands the ubiquitination and forms cross-links that render the ubiquitin cloud resistant to harsh detergents. Additionally, Steinbach et al. also demonstrate that Rab5 is recruited to the LCV and remains associated for a considerable period.

Strengths:

This manuscript is very well written, with clear justification provided for experiments that make it very easy to follow along with the experimental logic. The figures have clearly been designed with much thought and are easy to interpret. Steinbach et al have also done a commendable job of addressing the previous reviewers' comments, even though some may suggest that some of these comments could be viewed as slightly unreasonable. This work would be of interest to both the Legionella and ubiquitin fields. Legionella researchers would potentially be interested to explore the proposed barrier model as the function for the ubiquitin "cloud," whereas ubiquitin researchers may be interested in exploring the mechanisms underlying SidE's crosslinking ability.

# Referee report: Reviewer #2 (Public review)

**DOI:** 10.7554/eLife.108254.3.sa2

Summary:

The manuscript "Canonical and phosphoribosyl ubiquitination coordinate to stabilize a proteinaceous structure surrounding the Legionella-containing vacuole" by Steinbach et al. is well written and presents strong evidence that satisfactorily supports the main hypothesis and research objectives. The authors have clearly demonstrated the presence of cloud-like, detergent-resistant GTPase Rab5 surrounding the LCV, and formation of the structure is dependent on the SidE family of effectors. The study provides insights into the relevant (associated with described phenotype) ubiquitination pathways. The findings advance our understanding of *Legionella pneumophila* vacuole remodeling during intracellular infection and open directions for future research to establish broader implications of this structure on Legionella pathogenesis.

Strengths:

The manuscript convincingly demonstrates the presence of a cloud-like, detergent-resistant GTPase Rab5 surrounding the LCV through elegant microscopy. The experimental evidence about the dependence of the observed phenotype on the SidE family of effectors is compelling and presented with strong scientific rigor. The introduction is well-written, and the discussion is thorough and satisfactory. The article is thought-provoking and shows preliminary evidence for ubiquitin-mediated protection and spatial organization of the LCV.

# Referee report: Reviewer #3 (Public review)

**DOI:** 10.7554/eLife.108254.3.sa3

Summary:

This manuscript by Mukherjee and colleagues extended earlier studies on the coordination of the SidC and SidE effector families on the generation of a unique ubiquitin layer on the surface of the vacuoles containing the bacterial pathogen *Legionella pneumophila* (LCV).

Strengths:

The main strength of the manuscript is the identification of the small GTPase Rab5 as a major "carrier" of these differently modified ubiquitin and ubiquitin chains, which was nicely quantified.

Weaknesses:

The results are mostly descriptive, based on mechanistic studies from earlier works.

# Author comment: Author response

**DOI:** 10.7554/eLife.108254.3.sa4

**Authors:** Adriana Steinbach, Chetan Mokkapati, Puspangana Singh, Shaeri Mukherjee

**Affiliations:** University of California, San Francisco San Francisco United States; University of California, San Francisco San Francisco United States; University of California, San Francisco San Francisco United States; University of California, San Francisco San Francisco United States

The following is the authors’ response to the original reviews.

**Public Reviews:**

**Reviewer #1 (Public review):**

Summary:

In the submitted manuscript, Steinbach et al describe the formation of a detergent-resistant "cloud" around the Legionella-containing vacuole (LCV) that functions as a protective barrier. The authors show that formation of the "cloud" barrier is contingent upon the phosphoribosyl-ubiquitination activity of the SidE/SdeABC effector family, and is temporally regulated, with the assembly and subsequent disassembly of the "cloud" coinciding with replication and vacuolar expansion. The authors postulate a model of "cloud" barrier formation that relies upon a wave of initial ubiquitination by the SidC effector family, after which the SidE/SdeABC family expands the ubiquitination and forms cross-links that render the ubiquitin cloud resistant to harsh detergents. Additionally, Steinbach et al. also demonstrate that Rab5 is recruited to the LCV and remains associated for a considerable period.

Strengths:

This manuscript is very well written, with clear justification provided for experiments that make it very easy to follow along with the experimental logic. The figures have clearly been designed with much thought and are easy to interpret. Steinbach et al have also done a commendable job of addressing the previous reviewers' comments, even though some may suggest that some of these comments could be viewed as slightly unreasonable. This work would be of interest to both the Legionella and ubiquitin fields. Legionella researchers would potentially be interested to explore the proposed barrier model as the function for the ubiquitin "cloud," whereas ubiquitin researchers may be interested in exploring the mechanisms underlying SidE's crosslinking ability.

Weaknesses:

While the work is important and describes the physical nature of the ubiquitin cloud on the Legionella vacuole, it is somewhat descriptive in nature and does not dig deeply into what purpose this cloud serves. This is a complicated topic that will certainly stimulate additional research in this area.

We thank Reviewer #1 for positive assessment of our work. We acknowledge that our study leaves many mechanistic questions open and, as suggested by Reviewer #1, we hope that our data is thought-provoking for researchers studying *Legionella*, ubiquitin signaling, or both. We are greatly looking forward to the results of future experimentation on the role of the “cloud” surrounding the bacterial vacuole.

**Reviewer #2 (Public review):**

Summary:

The manuscript "Canonical and phosphoribosyl ubiquitination coordinate to stabilize a proteinaceous structure surrounding the Legionella-containing vacuole" by Steinbach et al. is well written and presents strong evidence that satisfactorily supports the main hypothesis and research objectives. The authors have clearly demonstrated the presence of cloud-like, detergent-resistant GTPase Rab5 surrounding the LCV, and formation of the structure is dependent on the SidE family of effectors. The study provides insights into the relevant (associated with described phenotype) ubiquitination pathways. The findings advance our understanding of *Legionella pneumophila* vacuole remodeling during intracellular infection and open directions for future research to establish broader implications of this structure on Legionella pathogenesis.

Strengths:

The manuscript convincingly demonstrates the presence of a cloud-like, detergent-resistant GTPase Rab5 surrounding the LCV through elegant microscopy. The experimental evidence about the dependence of the observed phenotype on the SidE family of effectors is compelling and presented with strong scientific rigor. The introduction is well-written, and the discussion is thorough and satisfactory. The article is thought-provoking and shows preliminary evidence for ubiquitin-mediated protection and spatial organization of the LCV.

Weaknesses:

The manuscript is well-organized and detailed, and it is hard to find weaknesses under the set goals of the research. A few weaknesses are that the molecular determinants or the regulatory mechanisms that drive selective versus non-selective incorporation of host proteins into this structure are unclear, and, as the authors mentioned, further work is required to establish the precise biophysical basis of the detergent resistance and expansive morphology of the ubiquitinated GTPase "cloud". Currently, the function or purpose of the structure is completely speculative. The effects or importance of the structure on bacterial replication is also not established in the current study. Figure 2D, right panel, Western blot results, the authors suggested the signal present in all four lanes between 37 and 25 kDa is 'nonspecific', which is probably a 'too intense' signal to be called so. Mass spec analysis would be interesting in order to identify sources of such intense signals. With these few limitations, the research presented in this manuscript is experimentally rigorous and opens avenues for future research.

We thank Reviewer #2 for their positive assessment and constructive criticism of our study. We agree that the degree of selectivity of incorporation of proteins into the “cloud” is of great interest, as are the molecular details of the cloud structure, and we expect that future experimentation in this area will provide insight into these key questions.

Reviewer #2 rightly points out that our study did not address the role of the LCV associated “cloud” in supporting bacterial replication. We note that previous studies have reported growth defects for knockout strains lacking SidC/SdcA (PMID 24483784) and the SidE family (PMID 27049943). However, given the multiple roles that these effector families appear to play during infection, we cannot ascribe these defects in bacterial growth solely to the absence of the LCV associated “cloud”.

As for the band present in the four lanes in Fig 2D, we suggest that this band is non-specific (most likely detection of the light chain of the antibody used for immunoprecipitation) because we do not observe this band in the input lanes, and we also see this band in the IP samples in Fig 2C (uninfected samples), including the vector control in which no PR-ubiquitination is observed. In Fig 2C, the non-specific bands in the IP samples appear lower intensity because the HA signal is relatively intense in comparison to the infection experiment in 2D, as overexpression of SidE family effectors results in far more PR-ubiquitination than in infection.

**Reviewer #3 (Public review):**

Summary:

This manuscript by Mukherjee and colleagues extended earlier studies on the coordination of the SidC and SidE effector families on the generation of a unique ubiquitin layer on the surface of the vacuoles containing the bacterial pathogen *Legionella pneumophila* (LCV).

Strengths:

The main strength of the manuscript is the identification of the small GTPase Rab5 as a major "carrier" of these differently modified ubiquitin and ubiquitin chains, which was nicely quantified.

Weaknesses:

(1) The results are mostly descriptive, based on mechanistic studies from earlier works.

(2) The majority of the work was dedicated to the characterization of the unique ubiquitin layer on the LCV. One important question was ignored: what is the role of Rab5 in this process? Is the GTPase activity of Rab5 required for its ubiquitination by SidC and SidE? The authors should create a Rab5 KO cell line, complement the line with different mutants of Rab5, and examine their ubiquitination and association with the LCV.

(3) The finding that Rab5 is associated with the LCV supports the notion that the LCV has characteristics of endo- or/late endosomes. The positioning of the LCV in the endocytic pathway should be discussed in the context of earlier studies (e.g.,PMID: 38739652; PMID: 11067875; PMID: 11067875).

We thank Reviewer #3 for their constructive criticism of our work. While we appreciate this reviewer’s interest in Rab5, our data is not consistent with Rab5 being a primary “carrier” of ubiquitin species; many more LCVs are ubiquitin-positive than Rab5-positive during early infection, and in our live imaging experiments we observe many ubiquitin-positive, Rab5-negative LCVs. We used Rab5 as a model substrate in this study because it allowed us to compare modification at the LCV membrane between the WT and avirulent *dotA* strain. Our data is more consistent with a model in which Rab5 is one of many small GTPases, and likely other host proteins, caught in a crosslinked mesh around the LCV. However, we agree that discussing the interaction of the LCV with the endolysosomal system is relevant; while this is discussed at length in our previous publication (PMID 38117589), we have expanded the discussion in this study to include new publications and contextualize our latest findings.

We agree with Reviewer #3 that assessing the role of nucleotide binding state in Rab5 ubiquitination is of interest. While creating a Rab5 KO cell line was not feasible given time and technical constraints, we conducted overexpression experiments with nucleotide binding mutants that exhibit dominant phenotypes (Q79L and S34N) and find that these mutants are still recruited to the LCV and ubiquitinated during infection (see new Figure 1 - figure supplement 1).

**Recommendations for the authors:**

**Reviewing Editor Comments:**

There are suggestions from the reviewers to further address the role of Rab5 in LCV-associated ubiquitination, including whether its GTPase activity is required for modification by SidC and SidE, and the mechanism underlying the dissolution of the ubiquitin cloud during vacuolar expansion.

**Reviewer #1 (Recommendations for the authors):**

To improve upon the manuscript and its impact, the authors could consider the following:

Major concern:

The temporal regulation of the ubiquitin cloud is fascinating. The authors nicely demonstrate that SidE- and SidC-type ligases cooperate to form the cloud, but how is it dissolved during vacuolar expansion? They demonstrate that ectopic expression of DopA can do this, but do DopA and DopB regulate this process natively?

We thank Reviewer #1 for their suggestions. While we agree that this line of experimentation is absolutely of interest, it is not feasible for our lab to carry out these experiments on a reasonable timeline to include in the current work.

Minor concern:

A syntax error on line 267 of the manuscript should be addressed.

This has been corrected.
